# Urea-based anticancer agents. Exploring 100-years of research with an eye to the future

**DOI:** 10.3389/fchem.2022.995351

**Published:** 2022-09-15

**Authors:** Roberta Listro, Giacomo Rossino, Federica Piaggi, Falilat Folasade Sonekan, Daniela Rossi, Pasquale Linciano, Simona Collina

**Affiliations:** ^1^ Department of Drug Sciences, University of Pavia, Pavia, Italy; ^2^ School of Pharmacy and Pharmaceutical Sciences, Panoz Institute, Trinity College Dublin, University of Dublin, Dublin, Ireland

**Keywords:** urea, anticancer drug, suramin, kinase inhibitors, patent survey, medicinal chemistry

## Abstract

Suramin was the first urea-based drug to be approved in clinic, and in the following century a number of milestone drugs based on this scaffold were developed. Indeed, urea soon became a privileged scaffold in medicinal chemistry for its capability to establish a peculiar network of drug−target interactions, for its physicochemical properties that are useful for tuning the druggability of the new chemical entities, and for its structural and synthetic versatility that opened the door to numerous drug design possibilities. In this review, we highlight the relevance of the urea moiety in the medicinal chemistry scenario of anticancer drugs with a special focus on the kinase inhibitors for which this scaffold represented and still represents a pivotal pharmacophoric feature. A general outlook on the approved drugs, recent patents, and current research in this field is herein provided, and the role of the urea moiety in the drug discovery process is discussed form a medicinal chemistry standpoint. We believe that the present review can benefit both academia and pharmaceutical companies’ medicinal chemists to prompt research towards new urea derivatives as anticancer agents.

## Introduction

### Suramin: The first urea-based drug. From trypanocidal to putative anticancer drug

**FIGURE 1:**
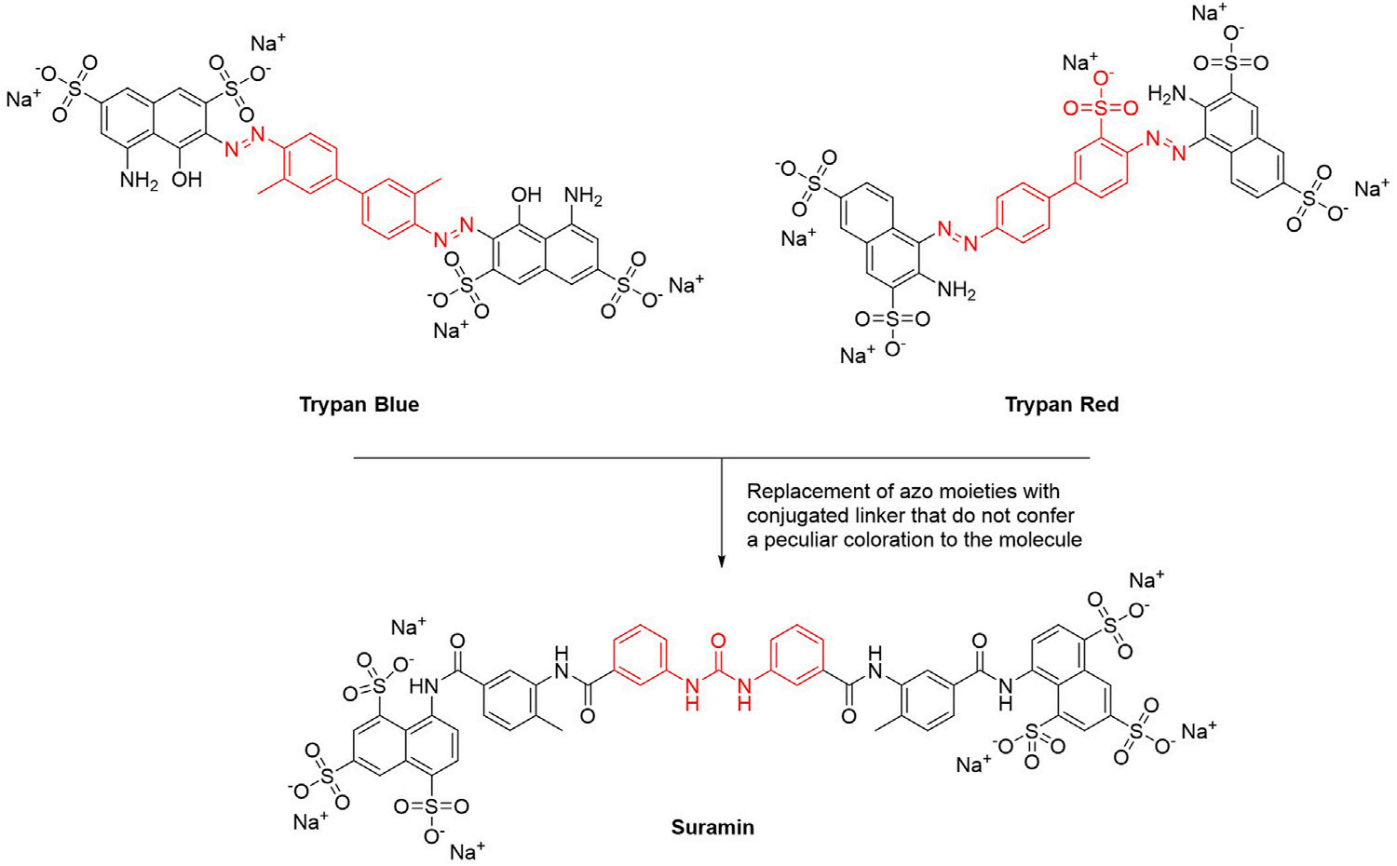
Chemical structures of suramin. The main structural modification that led to the discovery of suramin is highlighted in red.

This year marks the 100th anniversary of the introduction of suramin–the first chemotherapeutic agent developed in a medicinal chemistry project–to clinics (Wiedemar et al., 2020). Paul Elrich was the first to report the activity of dye or dye derivatives (such as trypan blue or trypan red) against tropical infections (Wainwright, 2010). To avoid skin staining as a side effect, and therefore looking for colorless derivatives, Oskar Dressel, Richard Kothe, and Bernhard Heymann at Bayer replaced the azo moieties present in trypan dyes with amido and ureyl linkers. These structural modifications preserved the conjugation between the aromatic rings but did not confer a peculiar coloration to the molecule. All urea derivatives showed higher antitrypanosomatidic activities compared to the original dyes. Particularly, suramin (molecule 205 in the original work) was the best-in-class compound and in 1922 it entered in therapy for the treatment of Human African Trypanosomiasis (HAT) (Figure 1) (Wainwright, 2010).

Despite its poor bioavailability and its intrinsic toxicity, suramin is still widely used to treat HAT (Steverding, 2010), and through the years, its use has been extended to other pathological conditions, including other parasitic infections (such as leishmaniasis and malaria) and viral infections (such as HIV, chikungunya, Ebola, dengue, Rift Valley fever and Zika), although with limited effects (Henß et al., 2016; Kuo et al., 2016; Tan et al., 2017). Starting from 1940s, suramin has been evaluated as an anticancer agent. At first, the capability of suramin in reducing the tumor mass in mice engrafted with lymphosarcoma was studied. Later, its potential anticancer activity was assessed in clinical trials against diverse neoplastic diseases such as melanoma, breast, prostate, bladder, brain, and lung tumors. None of these trials proved the efficacy of suramin as an anticancer agent in monotherapy (Parveen et al., 2022). Conversely, suramin was shown to be an effective chemosensitizer in *in vivo* models by enhancing the efficacy of other anticancer drugs such as cyclophosphamide, adriamycin, mitomycin C, taxol and carboplatin in mice (Osswald and Youssef, 1979). However, in a Phase I clinical study concluded in 2003, the association of nontoxic doses of suramin with taxol or carboplatin did not overcome drug resistance in patients with drug-resistant non-small cell lung cancer (Villalona-Calero et al., 2003). Several combination of suramin with anticancer agents endowed with different mechanisms of action have been proposed, namely protein kinases (Hensey et al., 1989), nucleic acid polymerases, fibroblast growth factor receptors (FGFR), heparanase (Nakajima et al., 1991), serine and cysteine proteases (Cadène et al., 1997), caspase (Eichhorst et al., 2004), telomerase histone- and sirtuin histone deacetylases (Trapp et al., 2007), methyltransferases (Feng et al., 2010), RNA-binding protein (i.e. HuR) (Kakuguchi et al., 2018), and many others. The association of suramin with estramustine and docetaxel showed promising results in hormone-refractory prostate cancer patients, though additional trials are necessary to warrant the clinical use of suramin in combinatorial antineoplastic therapies.

**FIGURE 2:**
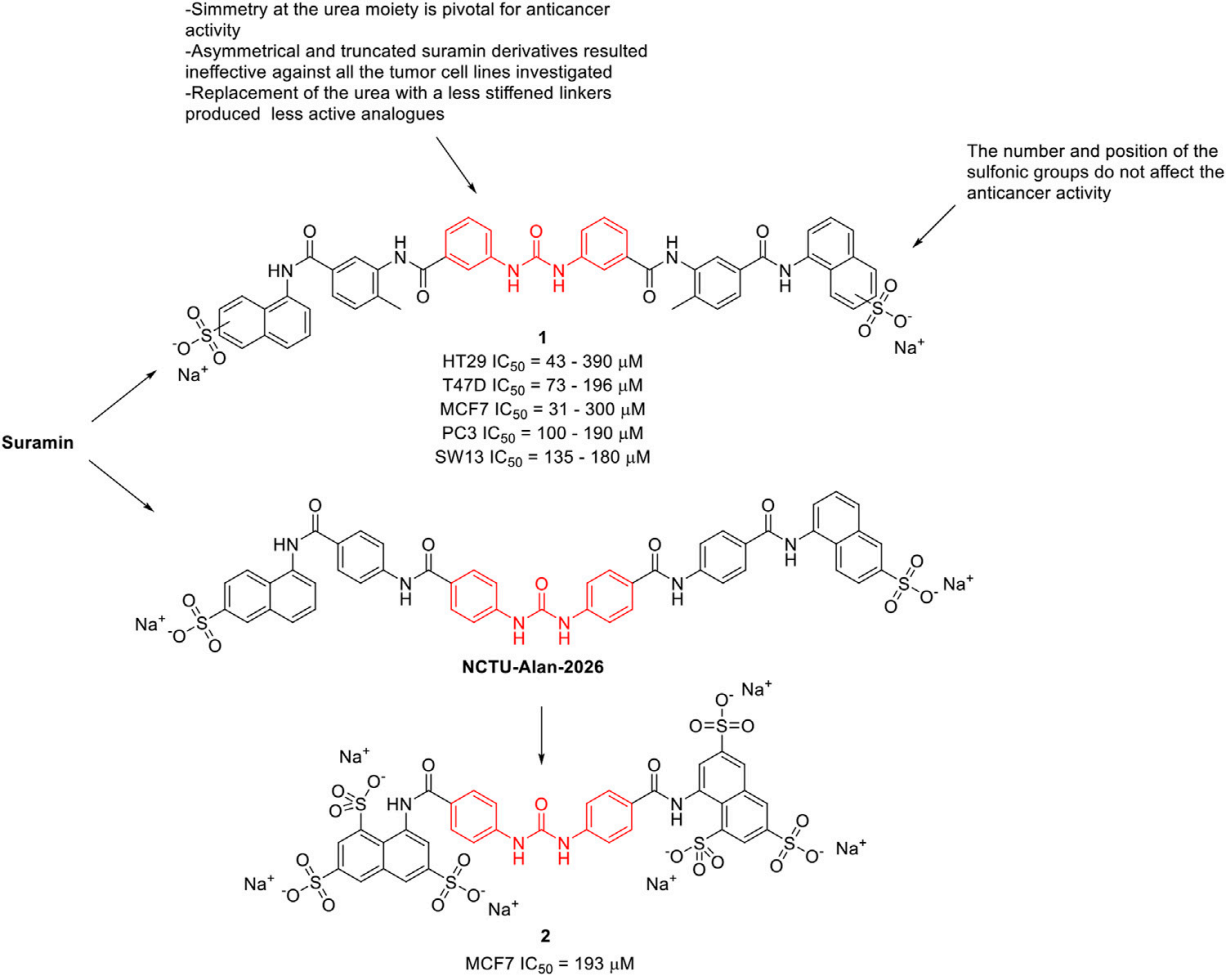
Chemical structures and SAR highlights of the suramin’s derivatives with proved anticancer activity.

Along the years, several suramin derivatives have been developed to enhance its anticancer activity. Firsching et al. (1995) were the first to investigate the antiproliferative and angiostatic activity of a series of nineteen suramin derivatives (**1**, Figure 2) against 5 different human cell lines (HT29, MCF7, SW13, PC3, and T47D). The structure activity relationship (SAR), mainly delineated on the HT29 activity, suggested that the number and position of the sulfonic groups do not affect the activity of the molecules (HT29 IC_50_ = 43–390 μM) indicating the effect of other functional groups, and in particular of the urea moiety, its antiproliferative activity. Indeed, the symmetry of the molecule around the urea moiety seemed to be mandatory for the anticancer activity, as the asymmetrical and truncated suramin derivatives were ineffective against the tumor cell lines that were investigated. Moreover, the replacement of the urea bridge with a less stiffened linker—succinic acid diamide—produced the least active of the suramin analogues (IC_50_ > 500 μM against almost all the selected cell lines). This trend was almost maintained against the other investigated cancer cell lines (T47D IC_50_ = 73–196 μM, MCF7 IC_50_ = 31–300 μM, PC3 IC_50_ = 100–190 μM, and SW13 IC_50_ = 135–180 μM). This evidence suggested the importance of the urea central unit for antiproliferative action (Firsching et al., 1995).

McCain et al. (2004) investigated the effect of suramin and 45 other congeners as inhibitors of protein-tyrosine phosphatases (PTPs), including Cdc25A. Cdc25A is considered an oncogene and it is overexpressed in breast, head and neck tumors. Several suramin derivatives resulted potent (IC_50_ < 5 μM) and specific (at least 20–30-fold specificity) Cdc25A inhibitors with respect to the other human PTPs tested. SAR profiles revealed that it was the core structure rather than the terminating functionality to affect the inhibitory properties of the molecules. It also appears that both halves of the symmetrical suramin core structure and urea moiety are required for high affinity binding to all PTPs studied. In 2020, Parveen et al. (2020) reported the antiproliferative activity of NCTU-Alan-2026 (Figure 2), a suramin analogue. The mechanism underlying NCTU-Alan-2026’s anti-mitogenic activity is mediated by the capability of the molecule to effectively interact with the heparan sulfate binding site of FGF1, thus blocking the interaction between FGF1 and FGF1R2 which ultimately results in anti-proliferative activity. The same authors designed and synthesized a new series of congeners and assessed the anti-proliferative activity in breast cancer MCF7 cell lines, with compound **2** (Figure 2) showing an IC_50_ of 193 μM. Although they were slightly less active than suramin (MCF7 IC_50_ = 153 μM), these derivatives showed a better safety profile, thus encouraging further *in vivo* investigation (Parveen et al., 2020). The binding of these compounds at FGF1 was confirmed by NMR spectroscopy.

## Urea-based anticancer drugs

**FIGURE 3:**
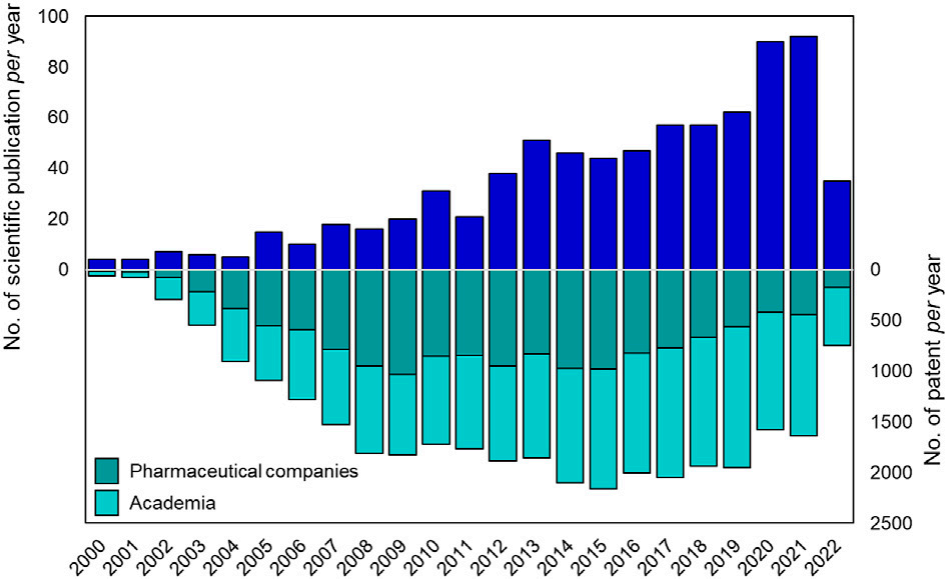
Number of publications (reviews and articles) and patents *per* year (from 2000 to 2021) reported in literature about urea-based compounds as anticancer agents. Source Scopus and Espacenet (last web access on 14 July 2022).

Suramin is the first ever drug containing a urea moiety to be introduced to clinics. Since then, urea soon became a privileged scaffold in medicinal chemistry, as proved by the presence of this moiety in several drugs and bioactive compounds endowed with a broad range of therapeutic and pharmacological properties. Querying the ChemBL, over 90,000 synthetic compounds possessing the urea scaffold in their structure were retrieved (Gaulton et al., 2012). Over the last 20 years there has been an increasing interest of the pharmaceutical community in the design and development of novel urea-based anticancer compounds as testified by the exponential growth in the number of documents published *per* year from the years 2000–2022 (Figure 3).

Recently, several comprehensive reviews have been published about the physicochemical properties of urea, the traditional and breakthrough synthetic methodology of this functional group, and the exploitation of this moiety in modern drug discovery and medicinal chemistry (Ghosh and Brindisi, 2020; Catalano et al., 2021; Liang et al., 2021; Siddig et al., 2021).

**FIGURE 4:**
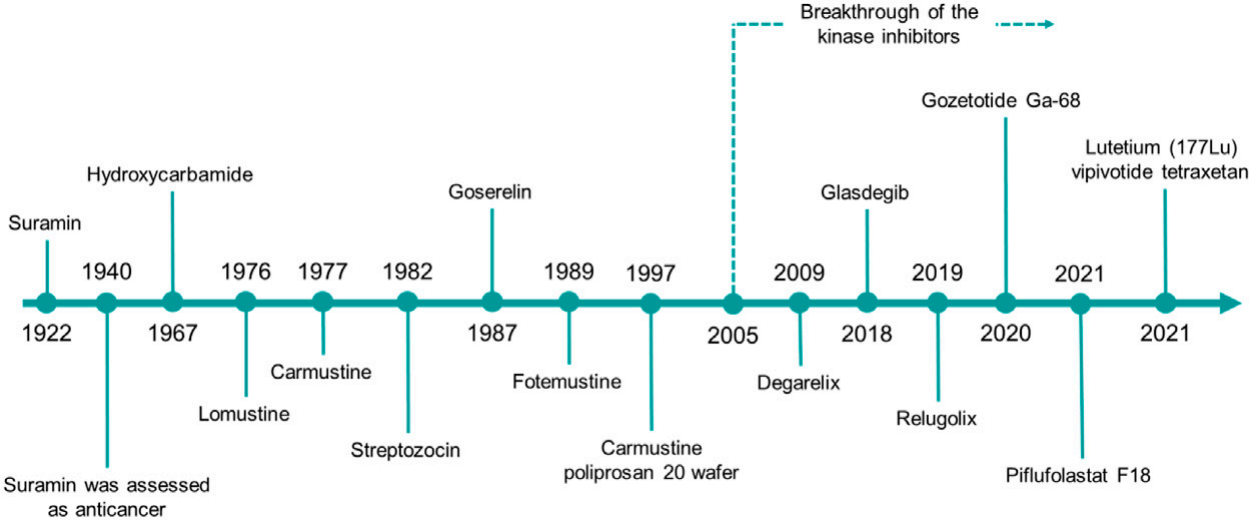
The timeline reports the year of the first approval in clinics of the urea-based anticancer drugs from 1922 to date.

**FIGURE 5:**
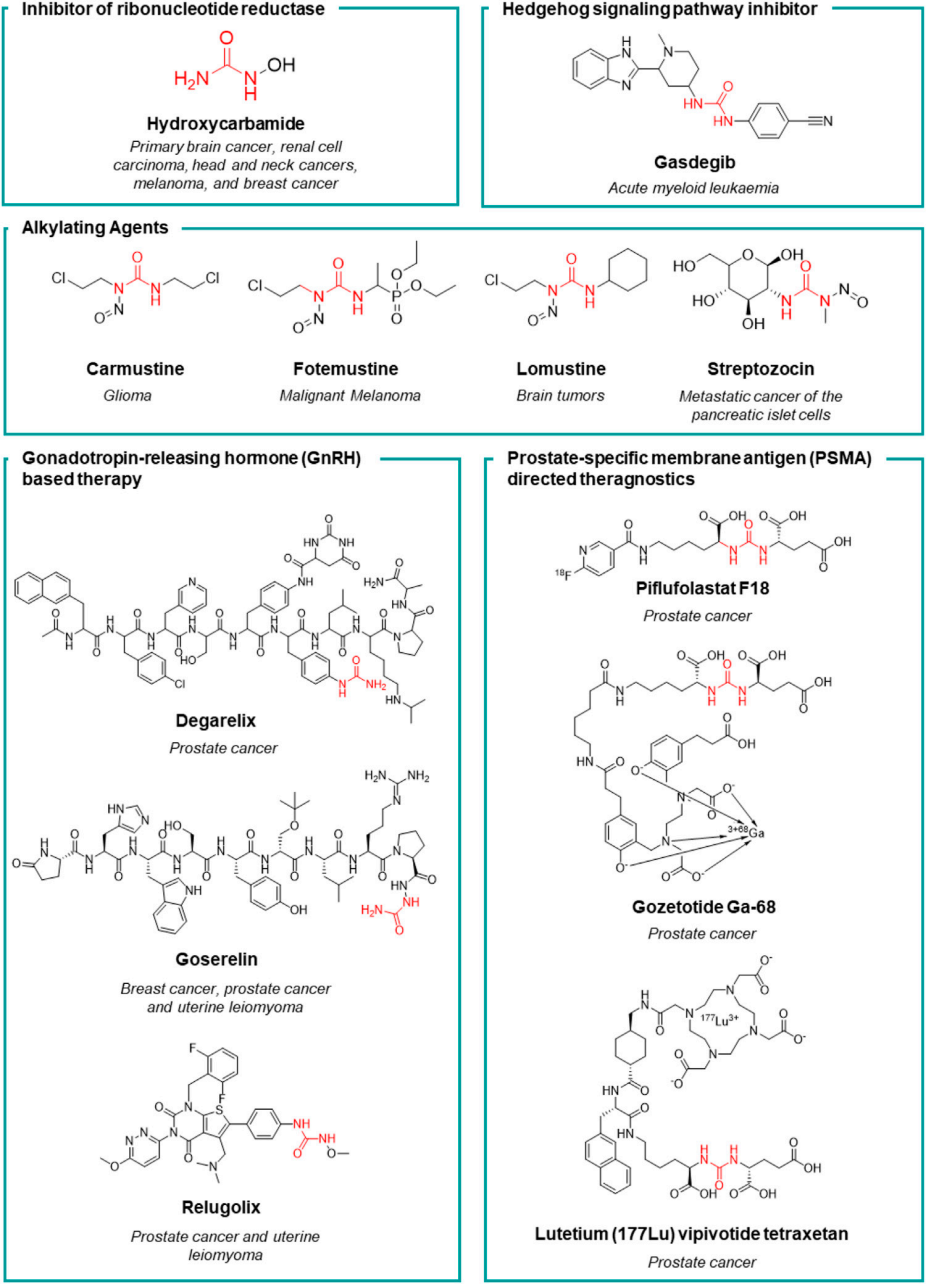
Chemical structure and main therapeutic use of the urea-based anticancer drugs (not active as kinase inhibitors) herein discussed.

Herein, we will give a comprehensive overview on the urea derivatives developed as anticancer agents, highlighting the relevance of the urea functionality from a medicinal chemistry standpoint, and providing the up-to-date information (according to FDA’s and EMA’s websites and to AdisInsight repository, last access on 16 August 2022) about their therapeutic use, and the highest clinical phase reached. We will provide a dissertation about urea-based anticancer drugs characterized by different mechanisms of action (Figures 4, 5), followed by a special focus on the kinase inhibitors approved for clinical use or currently in clinical trials. Lastly, a patent survey as well as an outlook on the future directions of the research in this field will also be drawn.

### Hydroxycarbamide

Hydroxycarbamide (HU, Figure 5) is an antimetabolite discovered in 1928, but its effectiveness as an anticancer drug was demonstrated in clinical trials in the 1960s by Bristol-Myers Squibb. In 1967, HU was launched worldwide for the treatment of diverse tumors such as primary brain cancer, renal cell carcinoma, head and neck cancers, melanoma, and breast cancer. Starting from 1980s, HU was further approved for the treatment of chronic myeloproliferative disorders (MPDs) and chronic myeloid leukemia (CML) (Spivak and Hasselbalch, 2011). HU is a selective and potent inhibitor of ribonucleotide reductase (IC_50_ = 87 μM) by selectively quenching the tyrosyl free radical. This induces the inhibition of DNA synthesis as HU starves the DNA polymerase at the replication forks for dNTPs (Koç et al., 2004). The non-specificity of its mechanism of action makes HU to be an effective cytostatic anticancer drug in a wide variety of cells with a high turnover. Moreover, the depletion of the deoxyribonucleotide pool induced by HU, enhances the anticancer activity of pyrimidine and purine antimetabolites. Several studies demonstrated the potential of HU in sensitizing tumors to other chemotherapeutic agents (Spivak and Hasselbalch, 2011).

### Alkylating agents

Nitrosoureas are an old class of alkylating anticancer drugs, characterized by the N-nitroso-urea scaffold from which their peculiar mechanism of action arises (Gnewuch and Sosnovsky, 1997). The N-nitroso-urea undergoes spontaneous degradation producing highly reactive alkylating species that generate DNA crosslinking, thus blocking DNA replication and transcription (Colvin et al., 1976; Kohn, 1977; Tong and Ludlum, 1978). Moreover, the reactive species may react with the nucleophilic residues of the DNA repairing enzymes causing their irreversible inactivation. Carmustine, lomustine, fotemustine and streptozocin are the main representative and still clinical used nitrosoureas (Figure 5) (Avendaño and Menéndez, 2015). **Lomustine**, was the first nitrosourea discovered and it was subsequently launched in 1976. Thanks to its high lipophilicity, it is able to cross the BBB by diffusion and for this reason it is mainly used for the treatment of brain tumors or as a second-line treatment for Hodgkin’s lymphoma. Lomustine inhibits the growth of U87 and temozolamide-U87 cell lines with an IC_50_ of 55 and 86 μM, respectively. Lomustine reduces the level of expression of the DNA repair protein O6-alkylguanine-DNA alkyltransferase (Yamamuro et al., 2021). Despite its congeners, lomustine is the sole nitrosourea that is administered orally. Lomustine is still approved in the EU but not in the United States. **Carmustine** was launched the following year (in 1977) and it is still used to treat several types of brain cancer including glioma, glioblastoma multiforme (U87 IC_50_ = 18.2 μM), medulloblastoma and astrocytoma, multiple myeloma, and lymphoma (Hodgkin’s and non-Hodgkin’s) (Avendaño and Menéndez, 2015; Kumar et al., 2020). **Streptozocin** is a natural anticancer antibiotic isolated from a strain of *Streptomyces achromogen*s. From a chemical standpoint it is a glycosylated nitrosourea and it was approved by the United States FDA in 1982 (Vavra et al., 1960 Bhuyan, 1970). The presence of the glucopyranoside sugar portion (in both anomeric forms) gives the structure good solubility in water in comparison to other lipophilic antineoplastic nitrosoureas. The sugar moiety makes the molecule selective for Langerhans cells as it exploits the glucose transporters to concentrate the compound in the β cells of the pancreas (Hosokawa et al., 2001). For this reason, it is mainly used for the treatment of metastatic cancer of pancreatic islet cells (Brentjens and Saltz, 2001). Lastly, **fotemustine** was developed by Servier and was launched for the first time in France in 1989. It has since been made available to other countries in the EU and worldwide, but not in the United States. Fotemustine alkylates guanine by forming chloroethyl adducts at the O6 of guanine, resulting in N1-guanine and N3-cytosine cross linkages, inhibition of DNA synthesis, cell cycle arrest, and finally apoptosis (Hayes et al., 1997). This agent is high lipophilic and crosses the blood-brain barrier and for this reason, it is employed for the treatment of disseminated malignant melanoma (IC_50_ = 173.326 and 125 μM against A375, MABe and RPMI-7591 cell lines, respectively), including cerebral metastases (Moarbess et al., 2008; Menaa, 2013).

### Gonadotropin-releasing hormone based therapy

**Degarelix** (Figure 5) is an injectable gonadotropin-releasing hormone (GnRH) receptor peptide antagonist launched in Europe in 2009 by Ferring Pharmaceuticals for the treatment of prostate cancer. Prostate cancer is known to be hormone-sensitive and responds to anti-androgen treatments (Chuu et al., 2011). Degarelix inhibits gonadal testosterone synthesis in men, and estrogen synthesis in women. It was developed within a medicinal chemistry program aimed at the amelioration of the pharmacodynamic and pharmacokinetic properties of the gonadotropin secretion antagonists cetrorelix (Bajusz et al., 2009) and ganirelix (Nestor et al., 1992). Despite their potency, these two peptides suffer from the stimulation of the release of histamine and relative short acting activity. Moreover, all the previously reported GnRH antagonists have poor water solubility and form gels at low concentration thus preventing the development of formulations that would last more than a month. To improve the solubility and the receptor binding ability of these peptides, functional groups that are able to form H-bonds such as the urea moiety (or cyclic congeners) have been introduced onto the side chain of specific amino acids. Degarelix was the most potent (GnRH IC_50_ = 3 nM) derivative, with very long-acting activity, higher solubility and without propension to form hydrogels, thanks to the presence of the urea moiety. It stabilizes a favorable peptide secondary structure that is less amenable to forming gels. Lastly, the chemistry of urea unlocked the synthesis of this peptide on solid support (Jiang et al., 2001). The improved pharmacodynamic and pharmacokinetic properties allowed for once monthly administration of the drug. Degarelix has been launched in the United States, EU, and other countries worldwide, for the treatment of prostate cancer. A single dose of degarelix, followed by a monthly maintenance dose, causes a rapid decrease in LH and FSH concentrations and, consequently, testosterone (Van Poppel, 2010). Serum concentrations of dihydrotestosterone (DHT) decrease in the same way as those of testosterone. The monthly maintenance dose allows for the suppression of testosterone to be sustained in 97% of patients for at least 1 year, well below medical castration levels (Persson et al., 2009).

**Goserelin** (Figure 5) is a synthetic analogue of the luteinizing hormone-releasing hormone (LHRH), developed by AstraZeneca. It has been launched in over 100 countries for the treatment of prostate cancer, early breast cancer, uterine hemorrhage, and endometriosis. Goserelin was discovered in 1987 when Dutta et al. synthesized a series of LHRH analogues containing an aza-aminoacid in position 6, 9, or 10 of the endogenous peptides. The introduction of an aza residue in the peptide was beneficial for obtaining derivatives with a higher affinity for the receptor site (GnRH IC_50_ = 2 nM) and for enhancing the stability towards enzymatic degradation, thus improving the pharmacokinetic profile (Dutta et al., 1978). Unlike degarelix, LHRH agonists induce a prolonged blockage of the production of androgen and estrogen hormones thus inducing a drastic reduction in the level of testosterone and estradiol after a month of therapy. An effect comparable to chemical castration is achieved after 4 weeks. As a side effect, such synthetic hormones may often stimulate the growth of prostate cancer in men, and breast cancer in women during the first 2 weeks of therapy, thus compromising the efficacy of the treatment (Nasser, 2022).

**Relugolix** (Figure 5) is a gonadotropin-releasing hormone (GnRH) receptor antagonist that prevents the secretion of GnRH (GnRH IC_50_ = 0.33 nM) (Huirne and Lambalk, 2001). This results in a reduction of the gonadotropins luteinizing hormone (LH) and follicle-stimulating hormone (FSH) levels, leading to the suppression of estrogen production in women and testosterone production in men. Low levels of estrogen and testosterone can help control of the growth of sex hormone-based tumors such as uterine leiomyoma in woman and prostate cancer in men. The research in this field led to the development of relugolix by Takeda. It was marketed in 2020 in the United States for the treatment of advanced prostate cancer (Shore et al., 2020). Relugolix was moreover registered also in Japan, for uterine leiomyoma (2019) and it was also approved this year in the European Union for the treatment of advanced hormone-sensitive prostate cancer. In addition, Myovant Sciences proved the beneficial effect of the co-administration of estradiol, norethisterone and relugolix for the treatment of heavy menstrual bleeding associated with uterine fibroids (uterine leiomyoma) (Ali et al., 2022). This drug association aims to optimize estradiol levels to achieve the long-term benefit of relugolix, while mitigating the side effects from a low-estrogen state. It is already available in the United States, and it has been approved in the EU for uterine leiomyoma.

### Hedgehog signal transduction inhibitors

**Glasdegib** (Figure 5), discovered by Pfizer, is the only drug currently approved for the treatment of acute myeloid leukemia (AML) and it represents the first and only Hedgehog pathway inhibitor approved for human use (Goldsmith et al., 2019; Thompson and Donald, 2020). This drug inhibits a transmembrane protein involved in hedgehog signal transduction (Fukushima et al., 2016). The Hedgehog signaling pathway plays an essential role in embryogenesis (Pak and Segal, 2016). Glasdegib was registered in 2018 by FDA for the treatment of AML in adult patients who are 75 years or older or have comorbidities that preclude the use of intensive induction chemotherapy (Goldsmith et al., 2019; Thompson and Donald, 2020).

### Prostate-specific membrane antigen directed drugs

**FIGURE 6:**
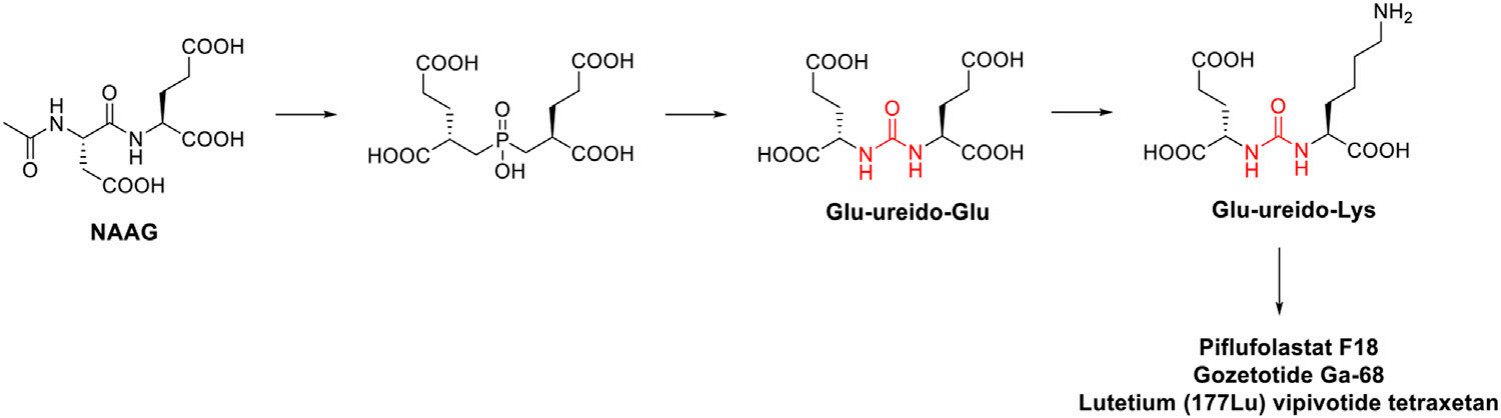
Medicinal chemistry rationale that was leading to the development of piflufolastat F18, gozetotide Ga-68 and lutetium (177Lu) vipivotide tetraxetan starting from NAAG.

Prostate-specifc membrane antigen (PSMA) inhibitors have been recently introduced in clinic as radiotracers or theragnostic agents. PSMA, also known as folate hydrolase I (FOLH1) and glutamate carboxypeptidase II (GCPII), is a 750-aminoacid type II transmembrane glycoprotein which is 1000-fold overexpressed on the surface of prostate cancer cells. This difference in expression of PSMA between normal and cancerous cells accounts for the high selectivity of these inhibitors (Evans et al., 2016). The discovery of these high affinity PSMA ligands, took place by Kozikowski et al. Starting from the structure of the N-Acetyl-L-aspartyl-L-glutamate (NAAG) peptide, which is one of the substrates of GCPII, the authors developed a first series of 4,4′-phosphinicobis-butane-1,3-dicarboxylic acids as potent GCPII inhibitor (IC_50_ = 21.7 nM). With the aim to develop a chemically accessible second series of congeners for a swift SAR study, the authors replaced the phosphinic moiety present in the first generation of PSMA ligands with the urea moiety (Figure 6). The urea was chosen to mimic a planar peptide bond between the two amino acids and to improve the resistance to enzymatic hydrolysis (Kozikowski et al., 2001). According to the results achieved, the glu-ureido-glu moiety first, and the glu-ureido-lys moiety then, began to become a common scaffold in the design of the PSMA ligands and of the corresponding radioactive-nuclide derivatives (Figure 6).

N-[N-[(S)-1,3-dicarboxypropyl] carbamoyl]-(S)-[^11^C]methyl-L-cysteine ([^11^C]DCMC) was the first reported radiolabeled inhibitor. However, the short half-life of carbon-11 forced the medicinal chemist to consider a different radioisotope, such as [^125^I] or [^18^F]. In particular, [^18^F] is a β+ emitting radionuclide that enables positron emission tomography. [^18^F]DCFBC (N-[N-[(S)-1,3- dicarboxypropyl]carbamoyl]-(S)-4-[^18^F]fluorobenzyl-Lcysteine) was initially synthesized as a proof of concept for the exploitation of fluorine-18 for clinical translation. Although [^18^F]DCFBC was suitable for the visualization of PSMA positive prostate cancer in mice, it is characterized by a long blood residence, which reduces the tumor-to-background ratio (Rowe et al., 2015). Conversely, the pyridyl derivative [^18^F]DCFPyL (2-(3-{1-carboxy-5-[(6-[^18^F]fluoro-pyridine-3-carbonyl)-amino]-pentyl}-ureido)-pentanedioic acid) showed a higher binding affinity for PSMA and tumor uptake, and lower blood persistence than [^18^F]DCFBC, with an overall suitable profile for use in humans (Chen et al., 2011). It was further developed by Progenics Pharmaceuticals under the name of **Piflufolastat F18** (Figure 5) and it is available in the United States for the identification of suspected metastasis or recurrence of prostate cancer. The candidate is under regulatory review in the EU. The extensive SAR around the glu-ureido scaffold, supported by the crystallographic structures resolved for four PSMA inhibitors, validated the urea as a worthy surrogate of the peptide bond. From X-ray structures, the glu-urea-lys moiety fits within the GCPII S1’ pocket with the same orientation for all the four derivatives, with the urea interacting with the active-site Zn_1_^2+^ ion and the side chains of Tyr552 and His553 (Barinka et al., 2008). Banerjee et al. designed a series of PSMA inhibitors based on the glu-urea-lys peptidomimetic that chelate ^68^Ga as radiotracers (Banerjee et al., 2010). [^68^Ga]PSMA-11 (also known as **Gozetotide Ga-68,** Figure 5) was the best-in-class derivative and was further developed by RadioMedix as a tomographic imaging enhancer radiopharmaceutical for the diagnosis of prostate cancer, adenoid cystic carcinoma, renal cell carcinoma, solid tumours, thyroid cancer, liver cancer and ovarian cancer, using positron emission tomography–computed tomography (PET-CT). Gallium (^68^Ga) gozetotide is approved in the United States for the diagnosis of prostate cancer and it is undergoing registration in several countries worldwide. However, Gallium (^68^Ga) gozetotide was not suitable for radiolabeling with therapeutic radiometals. To fix this issue, Benešová et al. reported the synthesis of 177Lu-PSMA-617, where the chelator was conjugated to the Glu-urea-Lys by a naphthalic spacer (Benešová et al., 2015). The naphthalic spacer introduced to reach the accessory hydrophobic pocket in the S1 pocket was detected through the analysis of the GCPII-PSMA inhibitor crystallographic structures. The naphthyl function improved the tumor-targeting and the pharmacokinetics of this PSMA inhibitor. 177Lu-PSMA-617 is characterized by a high binding affinity and internalization, together with a prolonged tumor uptake and rapid kidney clearance making it suitable for endoradiotherapy (ERT) (Pastorino et al., 2020). At the target site, the decay of Lutenium-177 releases a β-particle that causes the focused death of the cancer cell. 177Lu-PSMA-617 was further developed by Advanced Accelerator Applications (a subsidiary of Novartis) with the name of **lutetium (177Lu) vipivotide tetraxetan** (Figure 5). It is approved in the United States for the treatment of (PSMA)-positive metastatic castration-resistant prostate cancer. Preregistration is ongoing in EU.

### Kinase inhibitors

**FIGURE 7:**
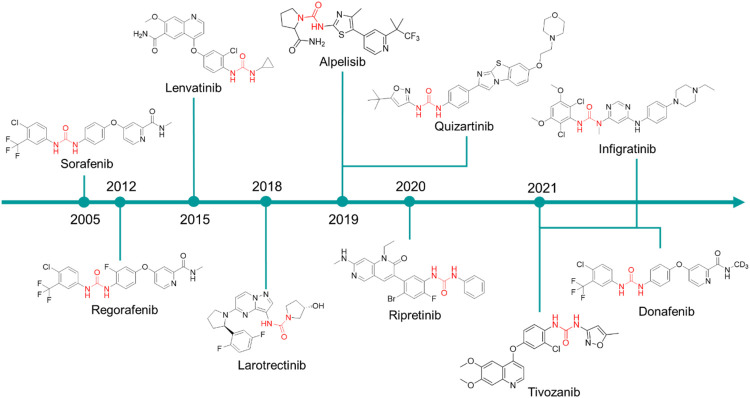
Chemical structure of the kinase inhibitors based on aryl-urea moiety approved for clinical use in humans from 2005 to 2021.

In the late 1990s, the discovery of the key role of the kinase-regulated biochemistry pathway in the genesis, sustainment and proliferation of cancer was a breakthrough for new, effective, and selective anticancer therapeutic strategies. Some kinases, such as EGFR (Ayati et al., 2020; Sabbah et al., 2020), VEGFR (Shibuya, 2011; Modi and Kulkarni, 2019; Wang et al., 2020), RAF kinases (Holderfield et al., 2014; Karoulia et al., 2017), PKC (Rui et al., 2017; Linciano et al., 2022), and Aurora kinases (Bavetsias and Linardopoulos, 2015; Yan et al., 2016), just to cite a few, are amplified in various cancer types and their dysregulation is associated with poor prognosis in cancers. Accordingly, the development of drugs targeting several kinases resulted in the winning pharmaceutical option (Kannaiyan and Mahadevan, 2018; Cohen et al., 2021). The approval of the first urea-based oral multikinase inhibitor for the treatment of cancer in humans (Sorafenib) in 2005 represented a milestone in this field. To ameliorate the pharmacodynamic and pharmacokinetic properties of sorafenib and to widen its activity against other tumors, several medicinal chemistry campaigns have been accomplished, leading to the discovery of over 60 kinase inhibitor anticancer drugs (Ayala-Aguilera et al., 2022). From a medicinal chemistry standpoint, the urea moiety played a pivotal role in the design of these kinase inhibitors, and it is present as a central scaffold in 10 kinase inhibitors over 66 approved drugs (Ayala-Aguilera et al., 2022). The urea-based kinase inhibitors are reported in Figure 7 and will be discussed in the following.

**FIGURE 8:**
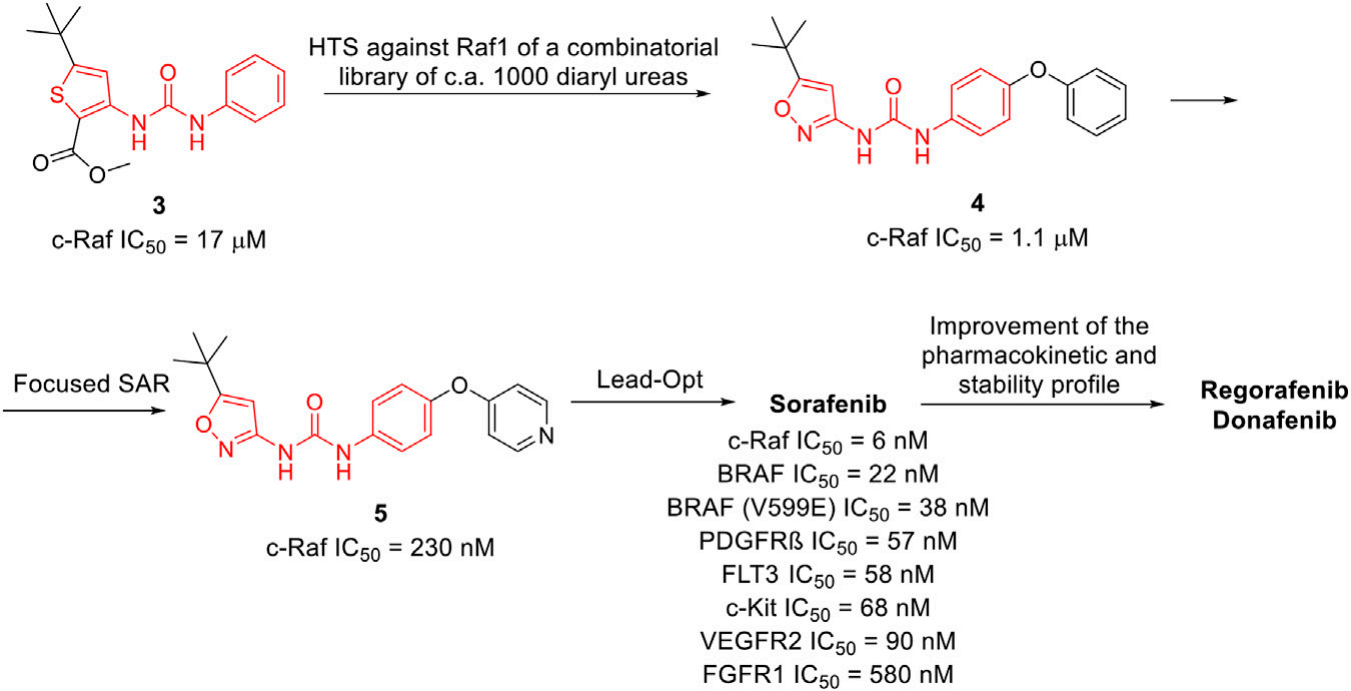
Key steps in the optimization process of sorafenib starting from the first identify diarylurea.

**Sorafenib** was discovered within a drug discovery campaign targeting the Ras–Raf–MEK–ERK oncogenic pathway (Wilhelm et al., 2006). The assessment of a library of 200,000 compounds against Raf1 (or c-Raf) kinase led to the identification of the 3-thienyl urea **3**, which exhibited a c-Raf IC_50_ of 17 μM and was therefore selected for further improvement (Figure 8) (Riedl, 2001; Smith et al., 2001). Nevertheless, the first SAR study around the 3-thienyl urea did not result in a significant improvement in the potency of the original hit compound. To quickly extend the SAR, both Bayer and Onyx Pharmaceuticals synthesized a new library of almost 1000 bis aryl ureas that were prepared using a parallel synthesis approach. The 3-amino-isoxazole **4** exhibited a c-Raf kinase IC_50_ of 1.1 μM (Figure 8) (Smith et al., 2001). The subsequent replacement of the distal aromatic ring with bioisosteres, while preserving the central urea moiety, led to **5.** It was selected as lead compound due to its druggability, its activity in HCT116 proliferation assays and its ability to decrease the phosphorylation of MEK and ERK. Further preclinical studies demonstrated the efficacy of **5** in inhibiting the growth of HCT116 xenografts, thus providing the first proof of concept for the inhibition of c-Raf kinase, a suitable anticancer therapeutic strategy (Figure 8). Further lead optimization studies confirmed the key role of the urea moiety in the Raf1 kinase inhibition. The modification of both the heterocyclic moiety and the distal pyridine ring of compound **5** led to the identification of **sorafenib** (c-Raf IC_50_ = 6 nM), which has been shown to be effective in both preclinical and clinical studies against several forms of human cancer (Lowinger et al., 2002). Further studies showed the capability of sorafenib to inhibit other intracellular (c-Raf, BRAF and mutant BRAF) and cell surface kinases (KIT, FLT-3, VEGFR-2, VEGFR-3, and PDGFR-ß) (Wilhelm et al., 2004).

The importance of the urea moiety in sorafenib and its congeners was rationalized in 2004, thanks to the X-ray crystallographic structure of BRAF in complex with sorafenib (Wan et al., 2004). As it can be observed in PDB ID: 1UWH, the distal pyridyl ring of sorafenib binds within the ATP binding pocket and interacts with Trp530, Phe582 and Phe594. The aliphatic side chains of Lys482, Leu513, and Thr528 contact the central phenyl ring of the inhibitor, whereas the trifluoromethyl phenyl ring is buried into a hydrophobic pocket delineated by helices C and E and the N-terminal portion of the kinase. The urea group that plays a pivotal role in c-Raf inhibition forms two hydrogen bonds; the first is established between the nitrogens of the urea and the carboxylate side chain of the catalytic Glu500, whereas the second involves the carbonyl moiety and the peptidic nitrogen of Asp593. These observations provided a clear rationale for the importance of the urea moiety in the design of effective c-Raf inhibitors (Wan et al., 2004).

**Regorafenib** is a fluorinated analogue of sorafenib (Wilhelm et al., 2011) with a comparable pharmacodynamic profile and an enhanced clinical performance, mainly related to the presence of the fluorine atom at the central aromatic ring. In 2012, it was approved the treatment of metastatic colorectal cancer (Dhillon, 2018) and in 2017 its use was extended to advanced hepatocarcinoma (Heo and Syed, 2018).

**Donafenib** is a modified form of sorafenib with a trideuterated N-methyl group at the distal pycolinamide moiety. The introduction of the deuterium atoms was guided by the necessity to potentially enhance the stability of sorafenib, thus improving the pharmacokinetic profile. *In vitro* studies demonstrated that donafenib and sorafenib have similar antiproliferative potency in multiple human cancer cell lines. Preclinical, Phase Ia and Ib studies have demonstrated good efficacy and safety profiles for donafenib (Bi et al., 2017; Li et al., 2020). Donafenib is approved in China for the treatment of hepatocellular carcinoma. Other clinical investigations for biliary cancer, acute myeloid leukaemia, thyroid cancer, gastric cancer, gastrointestinal cancer, esophageal cancer, nasopharyngeal cancer, colorectal cancer, cervical cancer and non-small cell lung cancer are underway in China.

**Lenvatinib** is another oral multikinase inhibitor successor of sorafenib. It is a type IIA inhibitor of VEGFR2 (IC_50_ = 4 nM), although Okomoto et al. recently suggested a re-classification of lenvatinib as a type V inhibitor (Okamoto et al., 2015). Lenvatinib is also an inhibitor of VEGFR1 (IC_50_ = 22 nM) and VEGFR3 (IC_50_ = 5.2 nM) and other pro-angiogenic and pro-oncogenic receptor tyrosine kinases, including FGFR 1 (IC_50_ = 46 nM), PDGFR α and β (IC_50_ = 51 and 39 nM, respectively), and KIT (IC_50_ = 100 nM). In 2015, it was approved for the treatment of non-radioiodine responsive thyroid cancer and in 2016 (Hamidi et al., 2022), for advanced renal cell carcinoma in combination with everolimus (Motzer et al., 2015, 2021). It is currently in clinical trials for the treatment of hepatocellular carcinoma. Lenvatinib differs from regorafenib and sorafenib as the diaryl-urea core scaffold has been replaced with an N-aryl-N-cyclopropyl urea. Notwithstanding this difference, the crystallographic structure of lenvatinib with VEGFR-2 demonstrated an overall comparable binding mode to sorafenib with BRAF (PDB ID: 3WZE) (Okamoto et al., 2015). Both drugs bind at the ATP-binding site of the receptor by exploiting the urea core scaffold which occupies a nearly identical position. The urea is involved in a network of H-bonds with the main-chain nitrogen of Asp1046 and the carboxylate in the sidechain of Glu885 as already observed in the binding mode of sorafenib at VEGFR-2. Notwithstanding the binding mode interests the ATP-binding site on VEGFR2 as well as the neighboring nonconservative allosteric region, the VEGFR2 structure complexed with sorafenib is in a DFG-out conformation, whereas VEGFR2 complexed with lenvatinib is in a DFG-in conformation. This different behavior might justify the longer residence time observed for lenvatinib than sorafenib (Okamoto et al., 2015).

**Tivozanib** is a potent pan-VEGFR tyrosine kinase inhibitor developed by Kirin Brewery, Co., Ltd. It showed picomolar activity against all three VEGFR isoforms (VEGFR-2 IC_50_ = 0.16 nM, VEGFR-l IC_50_ = 0.21 nM, and VEGFR-3 IC_50_ = 0.24 nM) (Nakamura et al., 2006). Additionally, it has nanomolar activity for cKIT and PDGFR at 1.63 and 1.72 nM, respectively. The sole information regarding the discovery of tivozanib is only reported in patent no. US2003087907A1 (Kubo et al., 2002). The medicinal chemistry aspect of this study is not available. Tivozanib was approved in 2017 by the EMA and in 2021 by FDA as a first-line therapy for the treatment of relapsed or refractory advanced renal cell carcinoma (Salgia et al., 2020).

**Ripretinib** is a novel type II tyrosine switch control inhibitor being developed by Deciphera Pharmaceuticals for the treatment of KIT or platelet derived growth factor receptor A (PDGFRA)-driven cancers, including gastrointestinal stromal tumor. Ripretinib inhibits KIT and PDGFRA kinases, including other kinases such as PDGFRB, TIE2, VEGFR2 and BRAF (Okamoto et al., 2015). Ripretinib is a type II “switch-control” kinase inhibitor that forces the activation loop (or activation “switch’’) into an inactive conformation (Smith et al., 2019). This innovative mechanism of action for ripretinib and congeners was confirmed by the recently resolved X-ray crystal structure of KIT1 kinase in complex with the ripretinib chloro analog DP-2976 (PDB ID: 3WZE). The carbonyl of the pyrimidone ring and the urea moiety in particular is involved in a dense network of H-bonds with the key amino acids Lys623, Asp640, and Glu810. These hydrogen bonds contribute to the switch of the kinase activation loop into the inactive type II state. Moreover, the pyridone ring forms additional hydrophobic interactions to further maintain the activation loop in its inactive state. The observed binding mode rationalized the peculiar mechanism of action of ripretinib. The sole information regarding the discovery of ripretinib is only reported in patent no. US8461179B1 (Flynn et al., 2012). The medicinal chemistry aspect of this study is not available. It was approved for medical use by the FDA in May 2020 and by the EMA in November 2021 for the treatment of patients with advanced gastrointestinal stromal tumors after previous therapy with kinase inhibitors.

**FIGURE 9:**
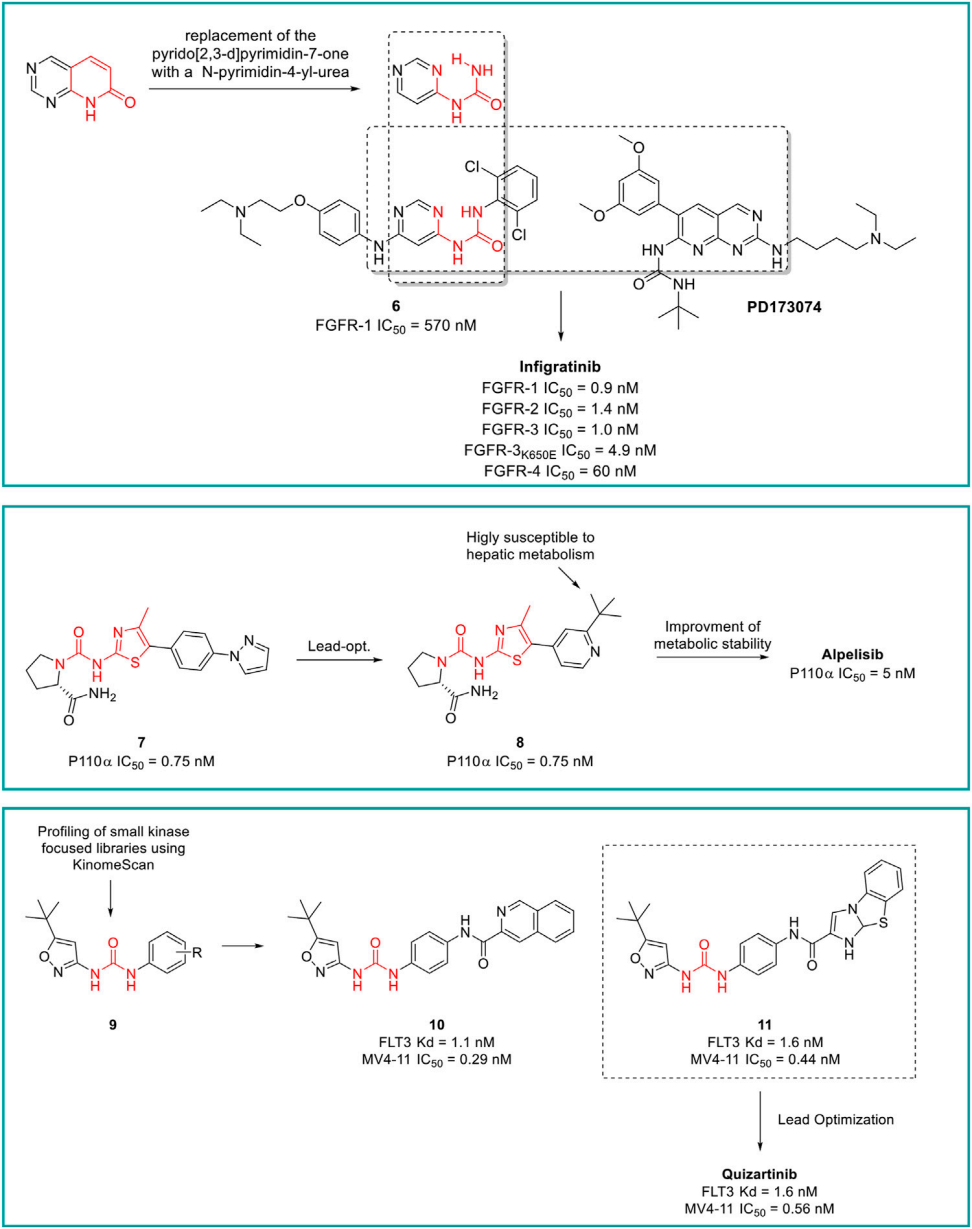
Key steps in the design of infigratinib, alpelisib and quizartinib.

**Infigratinib** is a pan-fibroblast growth factor receptor (FGFR) kinase 1-4 inhibitor, developed by Novartis. Aimed at the identification of potent and selective FGFR inhibitors, Guagnano et al. adopted a nonconventional strategy by replacing the pyrido [2,3-d]pyrimidin-7- one core structure of a well-known class of protein kinase inhibitors with a N-pyrimidin-4- yl-urea motif able to form pseudo six-membered rings by intramolecular hydrogen bonding, as depicted in Figure 9 (Guagnano et al., 2011). The first designed prototype compound **6** showed a submicromolar activity against FGFR1 (IC_50_ = 570 nM) (Furet et al., 2008). In a lead optimization process, the key structural motives of another FGFR1 inhibitor (**PD173074,** (Barvian et al., 2000)) were implemented in the chemical structure of **6**. Indeed, as depicted from the crystallographic structure of FGFR1 in complex with **6**, the two C (3) and C (5) methoxy groups on the aromatic ring of **PD173074** optimally fill the ATP hydrophobic pocket of the kinase (Mohammadi, 1998). A series of 3,5-dimethoxy-phenyl pyrimidinyl urea derivatives were prepared, with Infigratinib being the most potent and selective compound of these derivatives. It showed low/sub nanomolar activity against FGFR1/2/3/4 and high selectivity over a panel of 76 protein kinases. The achieved binary FGFR1-infigratinib complex validated the rationale behind the design of this new class of FGFR inhibitors. Infigratinib also possessed a suitable pharmacokinetic profile in rodents to support further *in vivo* investigation. It inhibited the proliferation of bladder cancer cells overexpressing wild-type FGFR3 and demonstrated significant anticancer activity in RT112 bladder cancer xenografts (Guagnano et al., 2011). In clinical trials, Infigratinib was assessed for the treatment of head and neck cancer, breast cancer, urogenital cancer, bladder cancer, glioblastoma, achondroplasia, gastric cancer, esophageal cancer, and solid tumors. It is available for the treatment of cholangiocarcinoma in the United States and is under regulatory review for the treatment of previously treated unresectable locally advanced or metastatic cholangiocarcinoma with a fibroblast growth factor receptor 2 (FGFR2) fusion or other rearrangement in the EU.

**Alpelisib** is an orally bioavailable phosphatidylinositol 3-kinase (PI3K) inhibitor that was developed by Novartis Oncology and assessed for the treatment of diverse solid tumors such as breast cancer, ovarian cancer, and head and neck cancer. Alpelisib is a selective inhibitor of PIK3 in the PI3K/AKT kinase signaling pathway (Yang et al., 2019). This signaling pathway is associated with cancerogenesis and its dysregulation may contribute to tumor resistance. Alpelisib was developed by the same team of medicinal chemists that in the same years discovered infigratinib. Furet et al., reported that the 2-aminothiazole scaffold was a suitable template for the design of selective PI3K inhibitors (Figure 9) (Furet et al., 2013). The first selective PI3Kα prototype (**7**) was obtained by appending a proline residue to the amino group in position 2 of the thiazole using a ureidic linker (Bruce et al., 2012). Through a structure-based approach, the molecule was properly modified to maximize the binding at the ATP pocket of PI3Ka without altering the ureido-thiazole core scaffold as the thiazole nitrogen and the 2-NH group formed key bidentate H-bonds with key amino acids of the kinase. All the designed derivatives showed low nanomolar inhibitor activity against PI3Ka, with compound **8** being the most interesting candidate. Unfortunately, metabolic studies on rat hepatic microsomes revealed a rapid metabolism of the compound, mainly at the t-butyl side chain. To fix this issue, one methyl group of the t-butyl radical was substituted with a CF_3_ which significantly improved the metabolic stability and reduced *in vivo* clearance, resulting in the identification of alpelisib. The crystallographic structure of alpelisib in complex with PI3Ka supported the medicinal chemistry rationale as the capability of the compound to establish a couple of H-bonds between the ureidic-thiazole and the side chain of Gln859 was experimentally observed (Furet et al., 2013). Alpelisib displayed a selectivity for p110a 50-fold higher than 442 other kinases, no inhibition of the CYP450 cytochrome and an excellent oral bioavailability in mice, rats and dogs. The proved anticancer activity of alpelisib in PI3Ka driven tumors in animal xenograft models and overall good tolerability supported the further clinical investigation (Furet et al., 2013). Interestingly, the combination of alpelisib and fulvestrant demonstrated increased antitumour activity in comparison to either treatment alone (André et al., 2021) and was registered in Australia and the EU in 2020 and marketed in the United States, Netherlands, Finland, UK and Canada in 2021.

**Larotrectinib** is a first-in-class, highly selective TRK inhibitor (Federman and McDermott, 2019). It was developed within a joint collaboration between Bayer and Loxo Oncology (a subsidiary of Eli Lilly), for the treatment of adult and pediatric patients with solid tumors (i.e., non-Hodgkin lymphoma, histiocytic disorders and primary CNS cancers) harboring neurotrophic receptor tyrosine kinase (NTRK) gene fusions (Yang et al., 2022). The sole information regarding the discovery of Larotrectinib is only reported in patent no. US8461179B1 (Flynn et al., 2012). The medicinal chemistry aspect of this study is not available. Larotrectinib binds at the ATP binding site of the TRK kinase family resulting in a potent inhibition of TRKA, TRKB and TRKC with IC_50_ values of 6.5, 8.1 and 10.6 nM, respectively, and in a highly selective manner over a panel of kinase and non-receptors (Federman and McDermott, 2019). TRK is mutated in a variety of cancer cell types and these mutations play an important role in tumor cell growth and survival and in drug resistance. Interestingly, larotrectinib showed nanomolar inhibitor activity against the most prevalent TRKA mutations (TRKA^G595R^ IC_50_ = 109.4 nM, TRKA^G667C^ IC_50_ = 32.2 nM, and TRKA^F589L^ IC_50_ = 43.1 nM). Larotrectinib was launched in 2018 in the United States for the treatment of NTRK gene fusions-based solid tumors and in the following years it was registered in the EU and other countries worldwide for the same therapeutic indication.

**Quizartinib** is an FLT3 kinase inhibitor developed by Daiichi Sankyo Company (previously Ambit Biosciences) and Astellas Pharma, for the treatment of acute myeloid leukaemia (AML) and myelodysplastic syndromes. FLT3 kinase is mutated in approximately one-third of patients with AML, and these patients are less responsive to traditional therapies. From a medicinal chemistry standpoint, quizartinib was developed starting from a high-throughput screening (HTS) of small libraries of compounds using the KinomeScan™ technology (proprietary of Daiichi Sankyo Company). Patel et al. fished out some urea derivatives with general structure **9** as potent FLT3 inhibitors (Figure 9). The aim of the SAR study was to explore the influence of diverse substituents on the aromatic ring on the FLT3 inhibitory activity, leading to the development of a series of derivatives with nanomolar IC_50_ values. Compounds **10** and **11** (Figure 9) were the most potent and selective FLT3 inhibitors (FLT3 IC_50_ of 1.1 and 1.6 nM, respectively) of the series with sub nanomolar IC_50_ in counteracting the MV4-11 cell lines proliferation and excellent pharmacokinetic properties and efficacy in a human tumor xenograft model in mice (Patel et al., 2009). Unfortunately, these two compounds suffer from very low aqueous solubility, especially at higher doses, which compromises the further development. Attributing their improper physicochemical properties to the presence of the amide moiety between the central aromatic ring and the distal heterocycles, Chao et al., in a lead-optimization program, removed the carboxamide and introduced diverse groups known to improve water solubility on the distal ring. The introduced substitutions on the main structure of **11** did not affect its potency and selectivity against FLT3 or its antiproliferative activity against MV4-11. Compound **12** was outstanding due to its superior pharmacodynamic (both *in vitro* and *in vivo*), pharmacokinetic and toxicological profile and was advanced into Phase II clinical trials for the treatment of acute myeloid leukemia, with the common name of quizartinib (Figure 9) (Chao et al., 2009). Quizartinib was marketed in Japan in 2019 and obtained pre-registration status in the United States and EU. However, the FDA and EMA express doubts on the approval of this drug due to the lack of a significant benefit-to-risk ratio (Garcia-Horton and Yee, 2020).

## The patent landscape

Urea-based compounds with potential anticancer applications have increased in number, as clearly demonstrated by the trend in the literature (Figure 3). A similar trend was observed for patent applications, highlighting the importance of this chemical scaffold in drug development at both industrial and academic level. In the last 20 years, more than 30 thousand patents regarding new potential anticancer agents have been published and among them, 890 patents are focused on urea derivatives as anticancer drugs.

In this section, patents filled in last 10 years are analyzed and discussed.

**FIGURE 10:**
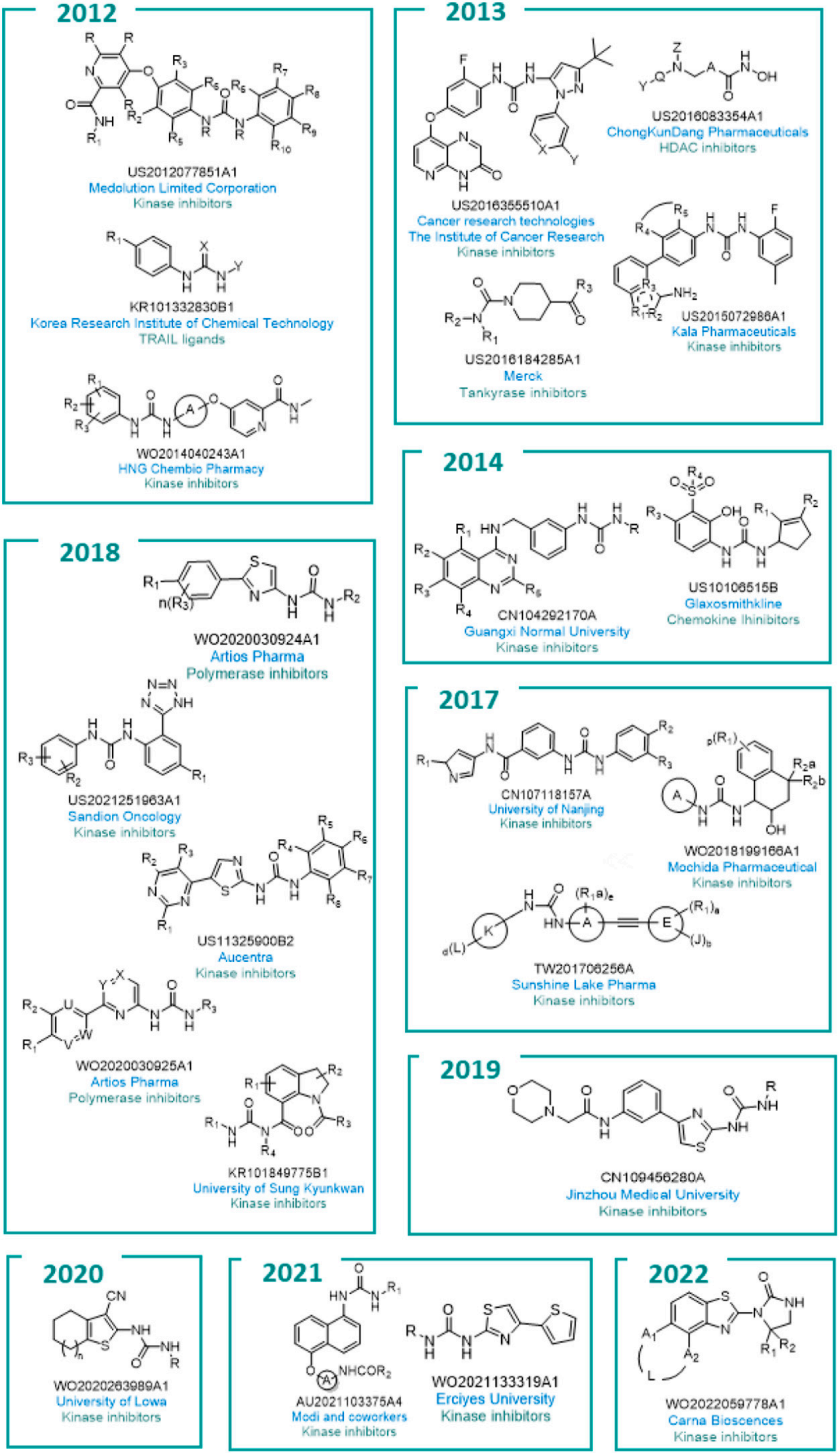
General structures of the different patented compounds.

All the general chemical structures of the urea-based molecules protected are reported in Figure 10.

**FIGURE 11:**
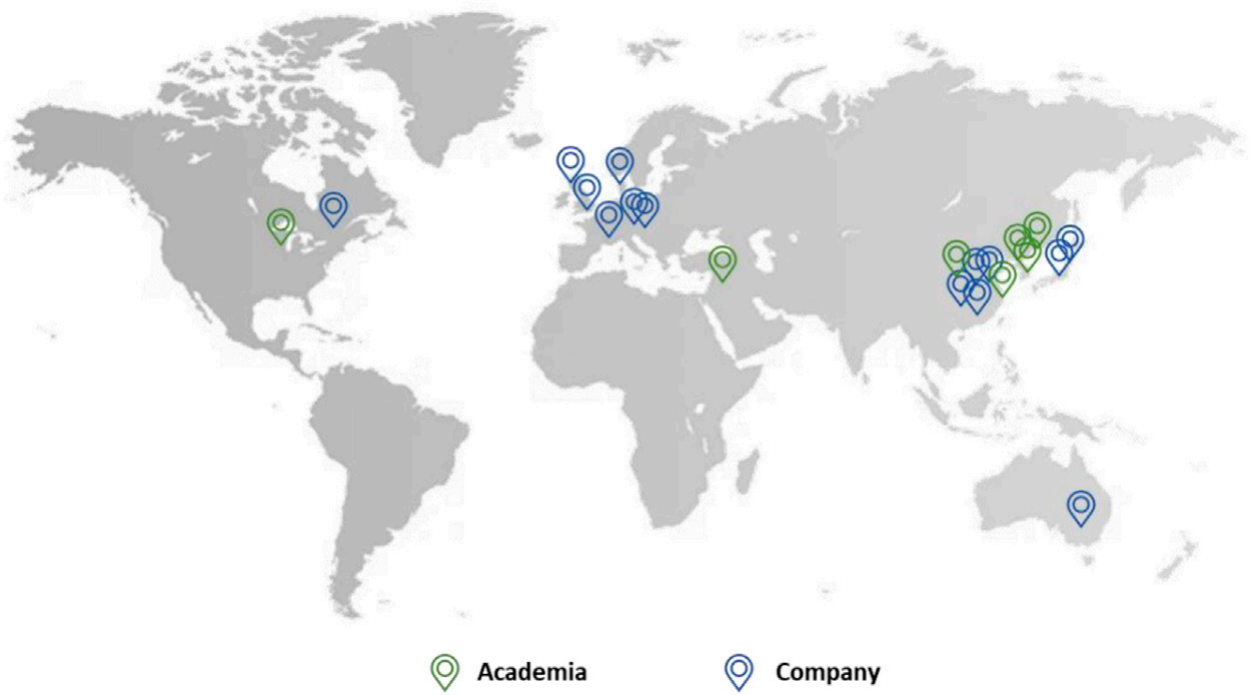
The location of academia (green) and companies (blue) which patented urea-based anticancer drugs worldwide.

Companies from all over the world in countries such as the UK, China, Japan, Denmark, Germany, and Australia are active in this field. This includes bigger industries such as GlaxoSmithKline and Merck as well as smaller companies such as Mochida Pharmaceutical and Aucentra (Figure 11).

Of note, since the exploitation of kinases as worthy anticancer targets is quite recent, and due to the discovery of new kinases involved in cancerogenesis, it is not surprising that the scientific world has focused its efforts on this hot topic. As a result, sixteen of the twenty-two patents cover the discovery of kinases inhibitors and ten are owned by Pharma companies.

In 2022, Carna Biosciences registered patent no. WO2022059778A1 (Cyclic urea derivatives) regarding the identification of selective dual-specificity tyrosine-regulated kinases (DYRK) inhibitors (Sawa et al., 2022). DYRK is a type of bispecific protein kinase that phosphorylates tyrosine, serine, and threonine. Five isoforms, namely DYRK1A, DYRK1B, DYRK2, DYRK3, and DYRK4, were identified to date (Boni et al., 2020). Particularly, inhibitors of DYRK1A are of interest for the treatment of EGFR-dependent cancer through the suppression of the cancer cells growing in EGFR-dependent tumors. The development of these derivatives is ongoing.

Scandion Oncology protected with patent no. US2021251963A1 (Urea derivatives for use in the treatment of subjects with elevated expression and/or activity of SRPK1) the discovery of new urea-based Serine/arginine-rich protein-specific kinase 1 (SRPK1) inhibitors as anticancer agents (Brünner and Jan 2021). SRPK1 is involved in the regulation of several mRNA processing pathways including alternative splicing, and overexpression of SRPK1 has been reported in multiple cancers including prostate, breast, lung, and glioma. Endovion (or NS 3728, SCO-101) was shown to be the most promising compound and is in Phase II clinical trials for colorectal tumor and in Phase I clinical trials for pancreatic and breast cancers.

Aucentra therapeutic registered patent no. US11325900B2 (5-(pyrimidin-4-yl)thiazol-2-yl urea derivatives as therapeutic agents) this year, regarding the discovery of urea derivatives as inhibitors of cyclin-dependent kinases (CDKs) (Wang et al., 2018). Of interest for its peculiar involvement in the physiopathology of cancer is isoform CDK8. CDK8 has specifically been reported to be an oncogene connected to colorectal cancer, activating β-catenin-mediated transcription that drives colon tumorigenesis. AU2-85 was presented as the most promising compound with proved anticancer efficacy and safety profiles in animal models. Currently, AU2-85 (or being a new chemical entity, it was renamed sunaciclib) is at the late stage of preclinical studies with a Phase I study planned for 2023.

Mochida Pharmaceutical registered patent no. WO2018199166A1 (Novel tetrahydronaphthyl urea derivatives) in 2017 (Saitoh et al., 2018). The company protected several urea derivatives represented by the general formula in Figure 10 as Tropomyosin receptor kinase A receptor (TrkA) inhibitors. TrkA is involved in several conditions such as pain, neurodegeneration, infection and particularly cancer. No information about clinical studies for the molecules of the present patent are reported at the moment.

In 2017, Sunshine Lake Pharma registered patent no. TW201706256A (Substituted urea derivatives and pharmaceutical uses thereof which provides substituted urea derivatives, and its stereoisomer, geometric isomer, tautomer, nitroxide, hydrate, solvate, metabolism product, ester, and pharmaceutically accepted salt or its prodrug), which was subsequently extended in Europe with accession no. US10065934B2 (Substituted urea derivatives and pharmaceutical uses thereof). In these patents, a deep investigation of the synthetic procedures for the preparation of urea derivatives as FLT3 inhibitors is reported (Cheng et al., 2017, 2018). FLT3 plays an important role in the proliferation and differentiation of hematopoietic stem cells. Overexpression of this receptor is found in AML (acute myeloid leukemia). The anticancer activity against MV4-11 cell lines expressing FLT3/ITD mutation was proved. None of the reported molecules are actually in clinical trials.

Three Pharmaceutical companies focused their efforts on the modulation of VEGFR. Kala Pharmaceuticals, HNG Chembio Pharmacy and Medolution Limited Corporation have patents nos. US2015072986A1 (Urea derivatives and uses thereof) (Kim et al., 2013), WO2014040243A1 (N-substituted phenyl-N′-substituted heterocyclic urea compound and application thereof as anticancer medicament) (Zhang et al., 2012), and US2012077851A1 (Urea derivatives as kinase inhibitors) (Zhang, 2012). HNG Chembio Pharmacy registered the design and synthesis of N-substituted heterocyclic urea derivatives with VEGFR-2 kinase inhibitor activity and the medical application as anticancer thereof. Medolution Limited Corporation protected a series of deuterated derivatives of sorafenib and regorafenib as multitarget kinase inhibitors for the treatment of VEGFR, PDGFR and/or RAF mediated cancer diseases (Zhang, 2012). Lastly, Kala Pharmaceuticals reported the synthesis of three different series of urea with potent and selective inhibitory activity against VEGFR. The most promising candidates are under preclinical studies for the treatment of cancer and ocular diseases (Kim et al., 2013).

In a public-private partnership, Cancer Research Technologies and the Institute of Cancer Research registered patent no. US2016355510A1 (1-(5-tert-butyl-2-aryl-pyrazol-3-yl)-3-[2-fluoro-4-[(3-oxo-4h-pyrido [2,3-b]pyrazin-8-yl)oxy]phenyl]urea derivatives as Raf inhibitors for the treatment of cancer) in 2013 (Springer et al., 2013). The advantages of targeting the inhibition of RAF as a cancer therapy were reported. RAF is a key downstream target for the RAS Guanine-nucleotide binding/GTPase proteins and also mediates the activation of the MAP kinase cascade consisting of RAF-MEK-ERK. RAF genes encode protein kinases that are thought to play important regulatory roles in signal transduction processes that regulate cell proliferation. The *in vitro* activity of the protected molecules, in terms of RAF inhibition was evaluated, along with the *in vivo* anticancer activity of the most interesting compounds.

Besides pharmaceutical companies, academia is also active in this field. In 2021, Modi S.J., Tiwari A and Kulkarni V.M. registered patent no. AU2021103375A4 (A rational Drug Design based identification of orally bioavailable 1,5-disubstituted naphthalene compounds as potent VEGFR-2 inhibitors) where three urea derivatives originating from different modification on sorafenib and regorafenib were reported as VEGFR-2 inhibitors (Modi et al., 2021). The most promising compound reduceed the cell viability of cancer cell line MCF-7 (breast adenocarcinoma), MDA-MB-231 (breast adenocarcinoma) and Hep G2 (hepatocellular carcinoma) and also suppressed the binding of VEGFR-2 on the surface of VEGF. The *in vivo* oral toxicity and pharmacokinetic properties were also investigated using Wistar rats.

Three academic patents focus on the discovery of inhibitors of a specific class of kinases named protein kinase enzyme activated by mitogen (MAPK). MAPK plays a key role in the signal transduction pathways of the p38 mitogen-activated protein kinase (p38MAPK) enzyme (Koul et al., 2013). Significant therapeutical results were attributed to p38MAPK inhibitors for the treatment of cancer, neuropathic pain, and periodontal diseases (Asih et al., 2020). The University of Nanjing registered patent no. CN107118157A (Design and synthesis of diphenyl urea derivative antitumor compounds containing pyrazol frameworks) in 2017, where the synthetic methods for the preparation of diphenyl urea derivatives with a pyrazol moiety and their effectiveness as antitumor agents against BRAF^V600E^ associated cancers were broadly investigated and protected (Zhu et al., 2017). Moreover, the abnormal activation of the downstream MEK-ERK signaling pathway which is crucial for tumor growth, proliferation, invasion, and metastasis was reported. With the same aim, the teams from the University of Iowa registered patent no. WO2020263989A1 (JNK inhibitors as anticancer agents) in 2020 (Salem and Elsaid, 2020). Herein, a series of ureas, benzamides and benzylamides derivatives were reported as c-Jun N-terminal kinase (JNK) inhibitors. JNKs are involved in several physiopathological processes, including inflammation, morphogenesis, and cell proliferation, differentiation, survival, and death. Persistent activation of JNKs is implicated in cancer development and progression. One of the main pathways controlled by JNK relies on the phosphorylation of the transcription factor p53. JNK is also implicated in the MAPK signaling cascade involved in cardiac hypertrophy. The urea derivatives were tested on A549 (lung carcinoma) cell lines using the MTS assay and showed promising activity. Lastly, in patent no. WO2021133319A1 of Erciyes University (Synthesis of urea derivatives which have p38 mapk inhibition and anticancer efficacy), which was registered in 2021, the inventors reported a series of urea-based compounds as effective inhibitors of MAPK (Dogan et al., 2021). Twenty-three urea derivatives were synthesized and their anticancer activity was evaluated through *in vitro* enzymatic inhibition assays. A real-time cell analyzer (xCelligence) system was adopted and performed on the most interesting compounds to monitor the cellular proliferation and inhibition of p38MAPK. Three urea derivatives were shown to be promising anticancer agents for further *in vivo* investigation.

Regarding the discovery of multi kinases inhibitors, of note is patent no. CN109456280A (4-Phenylthiazole-2-amine derivative containing urea structure, and preparation method and application thereof) that was registered in 2019 by Jinzhou Medical University. 4-phenylthiazole-2-amine ureas as anticancer agents were identified and protected (Cai et al., 2019). These compounds are multi kinase inhibitors that act on VEGFR, PDGFR-β, Kit, RET, Raf and Aurora, and showed promising antiproliferative activity against hepatic cancer cell lines with a comparable effect to sorafenib. University of Sung Kyunkwan registered patent no. KR101849775B1 in 2018 (N-N-aroylureas derivatives preparation method thereof and pharmaceutical compositions for the prevention and treatment of cancer containing the same as an active ingredient) (Kim and Sik, 2018). The inventors protected the discovery of novel N-aryl urea derivatives with anticancer activity against diverse human cancer cell lines characterized by an overexpression of different kinases, along with the development of a pharmaceutical composition for preventing and treating prostate and breast cancer. Lastly, in 2014, Guangxi Normal University registered patent no. CN104292170A (Quinazolinyl-aryl urea derivatives with antitumor function and application thereof) (JIANIAN et al., 2014). The synthetic procedure for the preparation of a series of congeners of sorafenib and gefitinib with quinazolinyl-aryl urea was reported. The compounds were assessed for *in vitro* kinase inhibitor activity and anticancer activity against several human cell lines.

Besides the exploitation of the urea moiety for the design of kinase inhibitors, this versatile scaffold inspired the development of anticancer small molecules that act on diverse targets involved in the physiopathology of cancer. In 2018, Artios Pharma Company registered two patents regarding urea derivatives as polymerase inhibitors. In patent no. WO2020030924A1 (Thiazoleureas as anticancer agents) the reported compounds were directed against RoIQ polymerase. RoIQ is a multifunctional enzyme that is comprised of an N-terminal helicase domain and a C-terminal low-fidelity DNA polymerase domain (Blencowe et al., 2020b). Both domains showed mechanistic functions in Microhomology-mediated end-joining (MMEJ). In general, RoIQ has been shown to be essential for the survival of homologous recombination-defective (HRD) cells and is up-regulated in HRD tumor cell lines. Accordingly, RoIQ is a valuable target for preventing or treating cancers such as breast cancer, ovarian cancer, prostate cancer, and pancreatic tumor retaining BRCA1 deficiency. In patent no. WO2020030925A1 (Heterocyclic substituted ureas, for use against cancer), Polq polymerase was selected as the target for a new series of heterocyclic urea derivatives (Blencowe et al., 2020a). PolQ is involved in the MMEJ mechanism, such as with the RoIQ enzyme. Inhibition of this enzyme might be exploited for preventing and treating tumor diseases, such as BRCA1 and BRCA2 deficient tumors, including breast, ovarian, prostate, and pancreatic cancer. The most interesting candidates in both patents are actually in Phase I or II for the treatment of metastatic solid tumors.

ChongKunDang Pharmaceuticals registered patent no. US2016083354A1 in 2013 (Novel compounds for selective histone deacetylase inhibitors, and pharmaceutical composition comprising the same) by which the identification of new compounds with histone deacetylase (HDAC) inhibitory activity was protected (Lee Changsik et al., 2013). Through the targeting of HDAC, the histone (protein) acetylation and chromatin structure were affected, inducing a complex transcriptional reprogramming exemplified by reactivation of tumor suppressor genes and repression of oncogenes. This target is particularly involved in several pathologies such as malignant tumors. Using Velcade as positive control, three compounds showed promising activity against multiple myeloma as pan HDAC inhibitors. Compound CKD-581 (or recently baptized as Alteminostat) is under Phase I studies for the treatment of lymphoma and multiple myeloma.

Lastly, among the others, the name of two important big pharmaceutical companies stand out. Glaxosmithkline reported in patent no. US10106515B2 (1-(cyclopent-2-en-1-yl)-3-(2-hydroxy-3-(arylsulfonyl)phenyl)urea derivatives as CXCR2 inhibitors) the exploitation of small molecule urea derivatives as anticancer agents targeting CXCR2. Indeed, chemokines regulate a broad spectrum of cellular functions and exert their actions by binding to chemokine receptors which are G protein-coupled receptors. CXCR2 is expressed on a variety of cells including neutrophils, keratinocytes, mast cells, eosinophils, macrophages, endothelial cells, and neurons, including sensory neurons. CXCR2 dysregulations are involved in carcinogenesis (Korbecki et al., 2022). The development of a medical system to improve drug-like properties was additionally reported.

Merck, however, exploited tankyrases as innovative targets for cancer diseases. In 2013, patent no. US2016184285A1 (Piperidine urea derivatives) was registered, which protected the synthesis and anticancer activity of new urea derivatives targeting Tankyrases 1 and 2 (TANKs) (Buchstaller and Dieter, 2013). The piperidine urea derivatives were assessed in an *in vitro* assay, for autoparsylation, measurement of cellular inhibition of Tankyrase through Axin 2 level, and ELISA Assay Biochemical Activity Testing of TNSK 1 and 2 studies. Korea Research Institute of Chemical Technology registered patent no. KR101332830B1 in 2012 (A Cancer sensitizer comprising phenylurea derivatives or salts thereof) which describes the development of phenyl urea derivatives as modulators of the tumor necrosis factor-related apoptosis (TRAIL) (Kim Seong, 2013). TRAIL belongs to the tumor necrosis factor (TNF) protein family, whose activation selectively induces the death of cancer cells by the formation of the death-inducing pathway complex, DISC and the autocatalytic activation of caspase 3. The urea derivatives reported in the patent have the capability to promote the expression of death receptors (DRs) and to effectively suppress the expression of anti-apoptotic proteins in a cancer cell line with TRAIL resistance.

Lastly, in 2022 the University of Pavia and University of Milano Bicocca filled the patent application no. EP21201359 (Substituted vinyl piperazine-piperidine urea derivatives as anticancer agents) (Collina et al., 2022).

A summary of the analyzed patents is reported in Table 1.

**TABLE 1:** Summary of cited patents.

Patent number	Patent applicant	Filing date	Mechanism of action	Development stage
KR101332830B1	Korea Research Institute	2012	TRAIL ligands	Preclinical phase
US2012077851A1	Medolution Limited Corporation	2012	Multi Kinases Inhibitors	Preclinical phase
WO2014040243A1	HNG Chembio Pharmacy	2012	VEGFR-2	Preclinical phase
US2016355510A1	Institute of Cancer Research	2013	RAF Inhibitors	Preclinical phase
US2016083354A1	ChongKunDang Pharmaceuticals	2013	HDAC Inhibitors	Phase 1
US2015072986A1	Kala Pharmaceuticals	2013	VEGFR Inhibitors	Preclinical phase
CN104292170A	Guangxi Normal University	2014	Multi Kinases Inhibitors	Preclinical phase
US10106515B2	Glaxosmithkline	2014	CXCR2 Inhibitors	Preclinical phase
CN107118157A	University of Nanjing	2017	MEK-ERK Inhibitors	Preclinical phase
W O 2018199166A1	Mochida Pharmaceutical	2017	TrkA Inhibitors	Preclinical phase
TW201706256A	Sunshine Lake Pharma	2017	FLT3 Inhibitors	Preclinical phase
WO2020030924A1	Artios Pharma Company	2018	RoIQ polymerase Inhibitors	Phase 1
WO2020030925A1	Artios Pharma Company	2018	PolQ polymerase Inhibitors	Phase 2
US11325900B2	Aucentra therapeutic	2018	CDKs Inhibitors	Phase 1
US2021251963A1	Scandion Oncology	2018	SRPK1 Inhibitors	Phase 1 and 2
CN109456280A	Jinzhou Medical University	2019	Multi Kinases Inhibitors	Preclinical phase
WO2020263989A1	University of Iowa	2020	JNK Inhibitors	Preclinical phase
WO2021133319A1	Erciyes University	2021	MAPK Inhibitors	Preclinical phase
AU2021103375A4	Modi S.J. and coworkers	2021	VEGFR1 Inhibitors	Preclinical phase
WO2022059778A1	Carna Biosciences	2022	DYRK Inhibitors	Preclinical
EP21201359	University of Pavia and University of Milano Bicocca	2022	Undisclosed	Preclinical

All the innovations discussed so far represent remarkable contributions to stimulate the development of new anticancer treatments. The numerous examples retrieved from the recent patent landscape prove the extensive exploitation of the urea moiety as a privileged scaffold in the design and development of new chemical anticancer entities by both pharmaceutical companies and academic research groups.

## Future perspective and outlook on novel compounds

**FIGURE 12:**
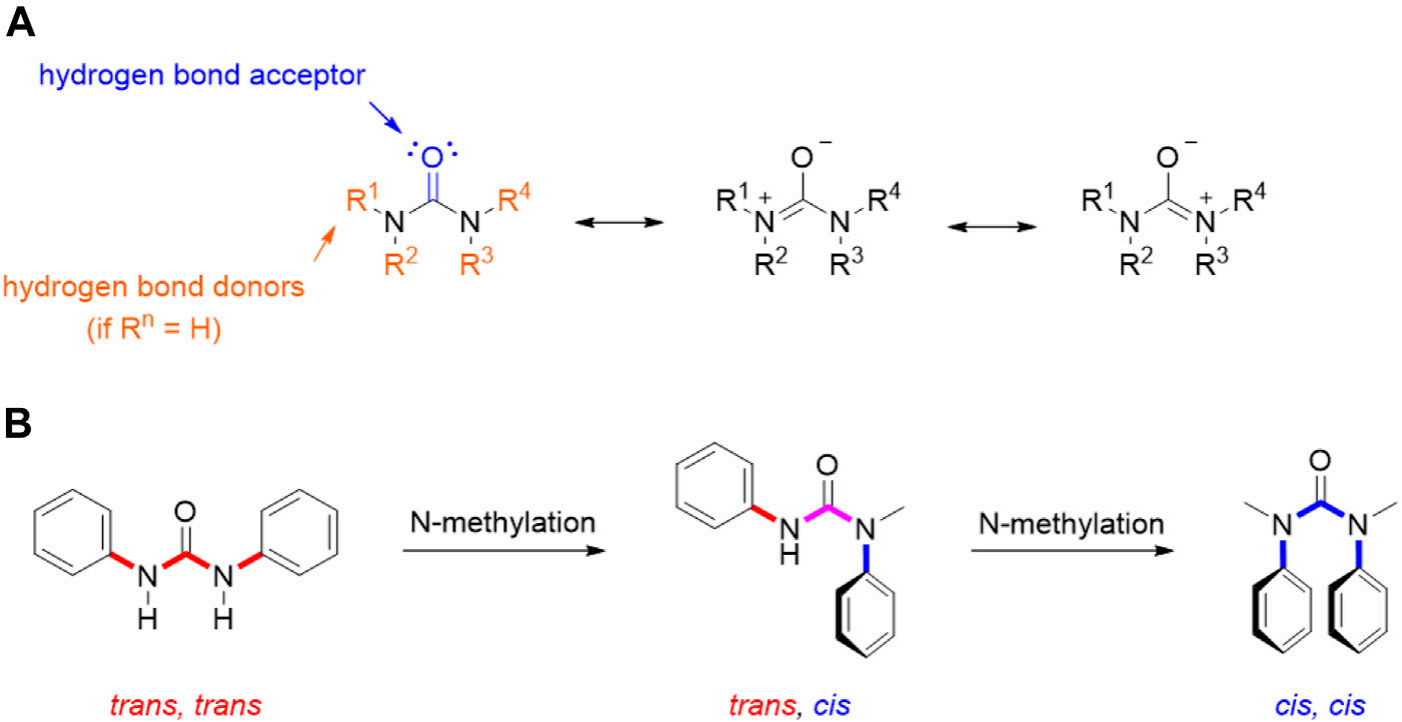
Resonance structure for urea moiety **(A)**; conformations of N,N′-diphenyl urea, N-methyl-N,N′- diphenyl urea, and N,N′-dimethyl-N,N′-diphenyl urea **(B)**.

As extensively reviewed above, many important antitumor agents contain a urea moiety, and the importance of this structural motif is further demonstrated by the large number of recent patents in this field. If we consider the unique properties of ureas, it is not surprising that this scaffold is regarded as a useful and versatile moiety from a medicinal chemistry standpoint. As described by the many examples reported in this review, the urea group can serve both as hydrogen bond acceptor and as hydrogen bond donor: the carbonyl group is a nucleophile and a hydrogen bond acceptor endowed with the possibility to engage two hydrogen bonds, whereas the two nitrogens, depending on the degree of substitution, can donate up to four hydrogens (in urea itself). Moreover, the three possible resonance structures of the urea generate a stronger negative dipole on the carbonyl and a positive dipole on the nitrogen than other carbonyl derivatives (Figure 12). This partial separation of charges contributes to the reinforcement of the H-bonds that this functional group is able to entangle with the biological targets.

This also entails some conformational restriction which affects the three-dimensional structure of urea derivatives. In fact, several studies have been conducted in order to determine their prevalent conformation in solution and solid state. Ganis P. and co-workers examined the structure of N,N′-diphenyl-N, N′-diethylurea through X-ray diffraction, demonstrating a non-planar distortion of approximately 30° of amide groups and a bond length of 1.37 Å for the C-N bonds (Ganis et al., 1970). Furthermore, the N-substitution on the urea moiety plays a key role in its conformation (Figure 12). Indeed, in the solid state, N,N′-diphenylureas assume a *trans, trans* conformation, which can be shifted to *cis, cis* by subsequent N-methylation (Figure 3). This conformation is characterized by the stabilizing π-π stacking interactions of the aromatic moieties. A dynamic state is shown in solution, even though the *trans, trans* isomer remains predominant. The possibility to switch the most populated conformation by applying simple structural modifications (e.g., N-methylation) is of great importance for tuning drug-target interactions as it can be exploited to rationally design conformationally constrained urea derivatives that better adapt, for example, to the binding pocket of a target protein, enhancing activity and/or selectivity (Lortie et al., 2003). The physicochemical properties of the urea functionality are of paramount importance in the drug design and development process, since they determine key parameters such as solubility, permeability, metabolism, and bioavailability.

Thanks to the hydrogen bond donor and acceptor features of the urea group, this moiety improves aqueous solubility and permeability. Urea derivatives were successfully employed as hydrotropic agents, i.e. compounds used to improve a drug’s solubility in pharmaceutical formulations (Cui, 2013; Herbig and Evers, 2013).

However, it is possible that ureidic compounds are not soluble in water and/or organic solvents. To overcome this problem with a medicinal chemistry approach, different strategies can be adopted: 1) modulation of hydrogen bonds; 2) disruption of planarity; 3) formation of a transient pseudo-ring structure. Regarding the modulation of the hydrogen bond, the introduction of electron donating and electron withdrawing groups on the nitrogen atoms allows for the modification of the inter- and intramolecular hydrogen bonds. Particularly, the nature of aliphatic moieties as nitrogen substituents establishes self-association of the molecules to promote the solubility in non-polar solvents (Lortie et al., 2003). Disrupting the planarity of the urea moiety reduces crystal packing energy and enhances water solubility. This can be achieved by introducing N-substituents to produce asymmetrical compounds (Ishikawa and Hashimoto, 2011). An additional method to disrupt planarity is the insertion of *ortho*-substituents on the N-aryl group of aryl ureas. A strategy that increases both solubility and permeability is the development of a transient pseudo-ring structure that involves intramolecular hydrogen bonds. In fact, the presence of a hydrogen bond acceptor near the urea moiety may lead the molecule towards a balance between closed (which increases permeability through lipophilic membranes via the reduction of exposed HBD) and open conformation (allowing the interaction of polar groups and solvent) (Alex et al., 2011). An example of this strategy can be found in the aforementioned FGFR inhibitor infigratinib, in which the stable pseudo-ring conformation mimics the pyrido [2,3-d]pyrimidin-7-one core of other known tyrosine kinase inhibitors (Guagnano et al., 2011). Furthermore, lipophilicity is of crucial importance for drugs targeting the central nervous system, as these should cross the blood-brain barrier by passive diffusion. Therefore, the modulation of the lipophilicity/hydrophilicity balance, exploiting the equilibrium between the open and close conformation and/or through the modification of N-substituents are other features accounting for the versatility of urea derivatives in medicinal chemistry.

**FIGURE 13:**
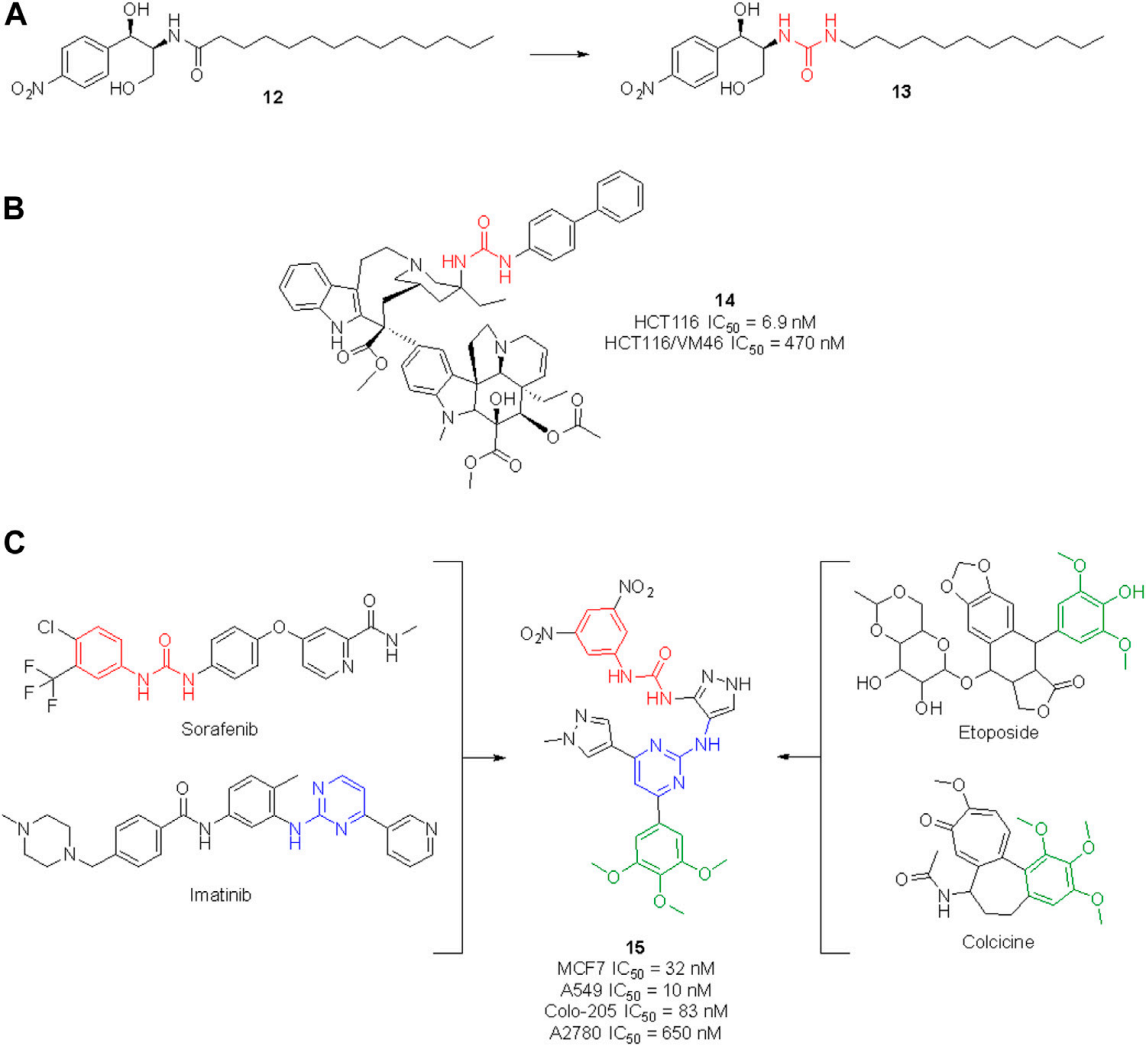
Development of new urea-containing ceramide analogs with anti-tumor activity **(A)**; structure of the vinblastine ureidic derivative **(B)**; design of a highly potent anticancer agent by merging the pharmacophoric elements of sorafenib, imatinib, colchicine and etoposide **(C)**.

The urea moiety has also been used as a bioisostere of amides (Kumari et al., 2020). For example, the ceramide analog **12**, which is endowed with potent cytotoxic activity against human colon cancer cells, underwent structural modifications and bioisosteric replacements to draw SAR considerations and identify novel anticancer agents (Figure 13). By substituting the amide group with a urea functionality, compound **13** was obtained, which emerged for its superior efficacy and broader activity toward other cancers (i.e., human renal, lung and prostate cancers as well as human leukemia) (Lim et al., 2003).

The high potential of the urea group to tweak the pharmacological profile of lead compounds encouraged further research in this field, and nowadays several medicinal chemistry research programs are taking advantage of this functionality to design novel molecules with an enhanced therapeutic effect. In the search for effective anticancer agents, natural products (NPs) have always been a major source of inspiration as privileged scaffolds endowed with enriched biological relevance (Martino et al., 2017, 2018; Khalifa et al., 2019; Malacrida et al., 2021; Varghese and Dalvi, 2021). Although the urea moiety is uncommon among secondary metabolites–i.e., compounds that are produced by living organisms to mediate ecological interactions–it can be added as a decoration or bioisotere on NP scaffolds. The vinblastine derivative **14** (Figure 13) represents a remarkable example of how the urea motif can be exploited to design semisynthetic NP derivatives with improved pharmacodynamic and/or pharmacokinetic profile in respect to the parent natural product. Vinblastine is a vinca alkaloid isolated from *Catharanthus roseus* used for the treatment of Hodgkin’s lymphoma, renal cell carcinoma, breast cancer, small cell lung cancer and colon cancer. Its mechanism of action involves the inhibition of microtubule assembly (Martino et al., 2017, 2018; Khalifa et al., 2019; Malacrida et al., 2021; Varghese and Dalvi, 2021). Leggans et al. reported a series of C20′ urea-based vinblastine derivatives and congeners with superior potency and activity against vinblastine-resistant tumor cell lines (Leggans et al., 2013). An extensive SAR revealed the importance of the urea moiety at C20′ over bioisosteric replacement with a thiourea, carbamate or amide. Interestingly, the introduction of a sterically demanding substituent on the second ureic nitrogen significatively contributed to the anticancer potency of these derivatives. These results contradicted the previous observation, i.e., that modification at C20′ of vinblastine might result in detrimental activity, opening instead new perspectives for the improvement of the anticancer activity of vinblastine derivatives. In particular, compound **14** showed a potency 10-times higher than vinblastine and promising cell inhibitory activity against the HCT116 (human colon cancer) and HCT116/VM46 (resistant human colon cancer) cancer cell lines (Leggans et al., 2013).

The growing interest of academia in this field is confirmed by the high number of very recent publications (i.e., within the last 2 years) concerning the discovery of novel antitumor agents containing the urea moiety.

For example, Cherukumalli at al. recently reported a series of new urea derivatives based on the pyrimidine-pyrazole core scaffold. These compounds were designed by combining the key pharmacophoric moieties of sorafenib, imatinib, colchicine and etoposide, as reported in Figure 13. The ten compounds were assessed for antiproliferative activity against several cancer cell lines (MCF-7, A549, Colon-205 and A2780) with IC_50_ values in the low/sub micromolar range. Compound **15** emerged for its excellent cytotoxicity activity, especially in comparison with etoposide. Molecular docking studies suggested that the anticancer activity could be derived from the inhibition of tubulin binding protein, human Abl tyrosine kinase and DNA topoisomerase. However, no experimental IC_50_ against these targets have been reported yet (Cherukumalli et al., 2022). Comparison of computational data with experimental results would allow for the development of an accurate SAR profile that assesses the role of the urea moiety of **15** in the interaction with the target(s).

**FIGURE 14:**
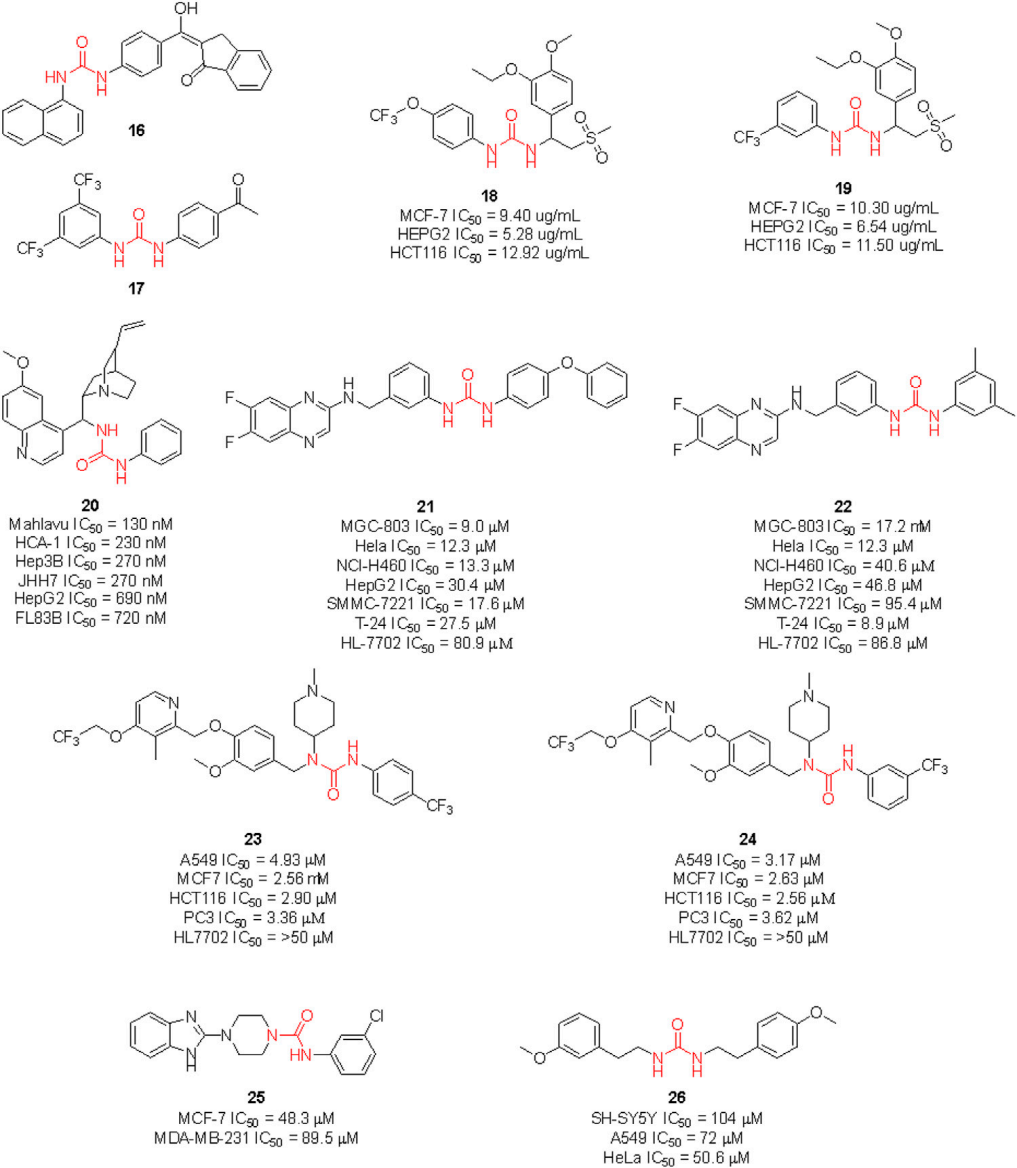
Urea-containing anticancer agents discovered in medicinal chemistry research programs within the last 2 years.

Gömeç et al. have very recently reported the synthesis of five diaryl urea-compounds with the aim to prove their anticancer properties against colon adenocarcinoma (Figure 14). Compound **16** showed promising anticancer activity against HT-29 cell lines with an estimated IC_50_ of 20 μM, with no cytotoxicity on healthy L929 fibroblast cell lines at the same dose. With a second aim to identify the putative target of **16**, docking calculations were performed against 34 key targets known to be involved in colon cancer. Briefly, **16** potentially being able to bind to Hsp90 revealed the importance of the urea moiety to effectively interact with the target. The urea forms three H-bonds with Gly97 and Lys58 involving the NH and the carbonyl, respectively (Gömeç et al., 2022). In a following study, the same authors reported the anticancer investigation of another set of diphenyl ureas. The designed compounds were predicted to target the estrogen receptor and were assessed for antiproliferative activity against the estrogen receptor (+) breast cancer cell line MCF-7. **17** was the most potent compound of the series with an estimated MCF-7 IC_50_ around 20 μM, but no cytotoxicity in healthy fibroblast cells (Gömeç et al., 2022).

Ganapathi et al., by replacing the phthalimide moiety of apremilast—which is considered a pharmacophore for PDE4 activity - with the aryl urea moiety, reported the design of a series of new sulfonylurea derivatives with anticancer activity. These compounds were tested against human breast (MCF-7), liver (HEPG2) and colon (HCT) cancer cell lines by SRB assay, using doxorubicin as a positive control. Compounds **18** and **19** were the most promising derivatives (Ganapathi et al., 2021).

Recent clinical evidence suggested the potential use of chloroquine (CQ) for the treatment of hepatocellular carcinoma (HCC) by exploiting the capability of this drug in blocking the autophagy. This occurred with a combination of CQ with sorafenib and only at high doses of CQ. Therefore, with the aim to design a dual molecule that can efficiently inhibit autophagy and induce cytotoxicity at the same time, Jin et al. synthesized a series of cinchona alkaloid derivatives with amide, squaramide, thiourea, and urea functional groups. Compound **20** was the most interesting derivative with sub-micromolar IC_50_ against diverse HCC cell lines (Figure 14). Moreover, it was proven to down-regulate Akt activation, reduce the antiapoptotic protein Bcl-xl expression and increase the activated caspase-3 in HCC cells and the expression of LC3-II and p62. The inhibition of autophagosome-lysosome fusion and suppression of the Akt/mTOR/S6k pathway in HCC cells was evaluated (Jin et al., 2021). Hence, this further supports the viability of the introduction of the urea moiety on NP scaffolds to identify new anticancer agents.

Li et al. (2020) synthesized a set of sorafenib derivatives by replacing the pyridine distal ring with a quinoxaline scaffold. The compounds were directly assessed for anticancer activity against six human tumor cell lines, namely MGC-80 (gastric mucinous adenocarcinoma), HeLa (epithelioid cervix carcinoma), NCI-H460 (cell lung cancer), SMMC-7721 (human papillomavirus-related endocervical adenocarcinoma), HepG2 (hepatocellular carcinoma) and T-24 (transitional cell carcinoma). Compounds **21** and **22** were the most interesting candidates with anticancer activity higher than 5-fluorouracil, cisplatin, and sorafenib against MGC-803 cell lines. Lower cytotoxicity on healthy HL-7702 cell lines was observed at the assessed doses. Although the molecules were designed as sorafenib derivatives, no inhibitor activity against kinases was reported.

Hou et al. reported another series of sorafenib derivatives by combining the diaryl urea core scaffold with the pyridyl moiety of proton pump inhibitors. Lansoprazole, indeed, was revealed to exert modest anticancer activity (Hou et al., 2021). The anticancer activity of the designed hybrid molecules was evaluated against non-small cell lung cancer (A549), breast cancer (MCF-7), colon cancer (HCT116) and prostate cancer (PC-3) cell lines. The best antiproliferative activities (IC_50_ < 5 μM), and low toxicity against healthy human liver normal HL7702 cell lines were observed for compounds **23** and **24**. Also in this case, no inhibitor activity against kinases was reported.

The above described glasdegib is an example of an already approved drug based on urea and benzimidazole scaffold. Looking for new promising anticancer compounds, Siddig et al. (2021) synthesized and assessed a series of novel thiourea-/urea-benzimidazole derivatives for cytotoxicity against MCF-7 and MDA-MB-321 human breast cancer cell lines. **25** was the most promising compound with IC_50_ of 48.3 and 89.5 μM against MCF-7 and MDA-MB-231 cells, respectively.

Lastly, Özgeriş et al. reported the synthesis of the unsymmetrical urea **26** and investigated its activity as an antibacterial, anticancer and antioxidant agent. Whilst **26** did not show antimicrobial activity, remarkable activity against SH-SY5Y (IC_50_ = 104 μM), A549 (IC_50_ = 72 μM), and especially against the HeLa cells (IC_50_ = 50.6 μM), was observed (Özgeriş et al., 2022).

The examples reported above are evidence that urea is an important and versatile moiety of the medicinal chemist’s toolbox: it can be exploited to engage key drug-target interaction (thus enhancing potency and/or selectivity) and to improve the pharmacokinetic profile of a given molecule, modulating water solubility, lipophilicity and metabolic stability. Moreover, it can be used as a bioisostere and to structurally modify natural products.

**FIGURE 15:**
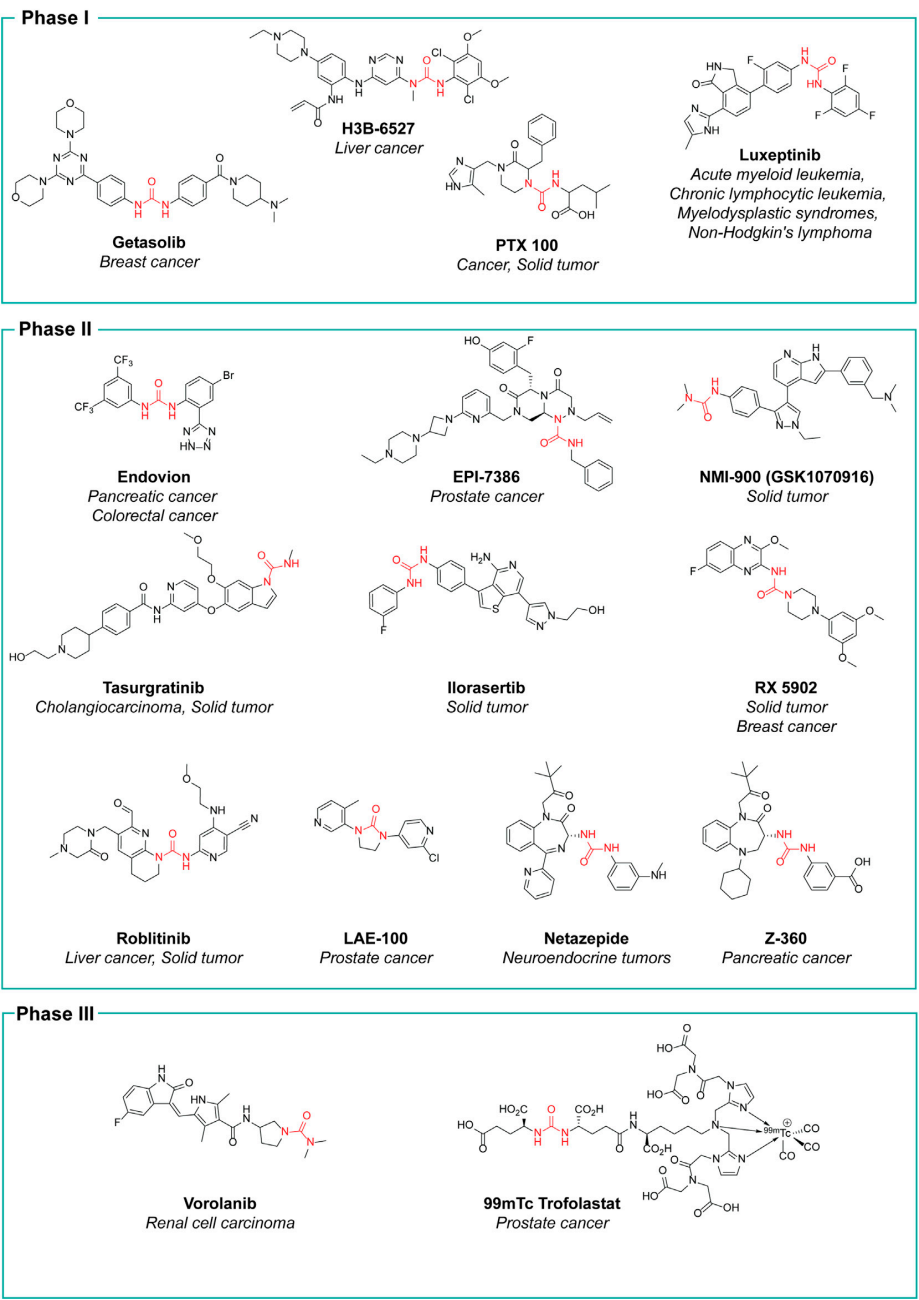
Urea-containing compounds currently under clinical evaluation as anticancer agents. For each drug candidate, the highest phase of clinical trials that has been attained is indicated, along with the specific therapeutic indication.

The research in the field is expected to increase, not only with the discovery of new hit compounds, but also by virtue of the numerous urea derivatives currently under clinical trials for the treatment of different types of cancer. New molecular entities that have not already been presented throughout this review, but are now under advanced evaluation are reported in Figure 15, along with their clinical study phase and therapeutic indication (source AdisInsight, last access on 14 July 2022).

Despite the significant advantages and the high versatility of the urea functionality, it must be noted that in some cases, urea derivatives can still present problematic or sub-optimal pharmacokinetic properties which cannot be overcome by structural modifications. In these cases, an approach based on pharmaceutical technology can be adopted. Even old active ingredients can have a new life thanks to innovative formulations that enhance their bioavailability and/or safety.

For example, the aforementioned carmustine can lead to severe side effects upon systemic administration, due to its high reactivity. To overcome this issue, in 1997, 20 years after the first approval, Arbor Pharmaceuticals developed and led the carmustine polifeprosan 20 wafer to the market for the treatment of glioma. The authorization was extended in Europe in 2008. This pharmaceutical specialty is a sustained-release biodegradable wafer implant that delivers the chemotherapeutic drug directly to the site of a brain cancer during the surgery, thus minimizing the drug-exposure to other areas of the body (Della Puppa et al., 2011). Very recently, a biosimilar of goserelin formulated as a monthly or trimonthly subcutaneous implant has been developed by Alvogen for the treatment of cancer, female infertility, uterine leiomyoma and endometriosis. An extended-release (XR) microsphere formulation of goserelin is being developed by Luye Pharma Group, utilizing its long-acting and extended-release technology platform for the development of the product. This goserelin formulation was preregistered for prostate cancer in China in 2021 and is in Phase III clinical trials for breast cancer.

A summary of the compounds in clinical trials is reported in Table 2.

**TABLE 2:** Clinical trial progression and therapeutic indication for the new chemical entities.

Type of cancers	NCT number	Candidate	Phase 1 Phase 2 Phase 3
Breast cancer	NCT03911973 and NCT04238715	Gedatolisib and Tasurgratinib	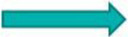
	NCT02003092, NCT01173913	RX5902, Docetaxel/Ritonavir	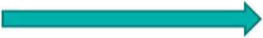
Prostate cancer	NCT04421222, NCT01173913 NCT04060394	EPI 7386, Docetaxel/Ritonavir and LAE 001	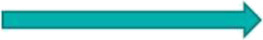
Pancreatic cancer	NCT03511222 and NCT00288925	Vorofonib and 1360	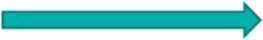
Solid Tumor	NCT03900442 and NCT03511222	PTX 100 and Vorolanib	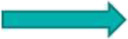
	NCT04238715, NCT02325739, NCT02003092, NCT01173913 and NCT02478320	Tasurgratinib, Roblitinib, RX5902, Docetaxel/Ritonavir and Ilorasertib	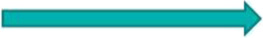
Liver cancer	NCT02834780	H38 6527	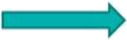
	NCT02325739	Roblitinib	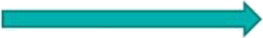
Acute myeloid leukemia	NCT03893682	Luxeptinib	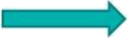
Non-small cell lung cancer	NCT03511222	Vorofanib	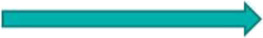
Chronic lymphocytic leukemia, Myelodysplastic syndromes, Non-Hodgkin’s lymphoma	NCT03893682	Luxeptinib	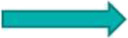
Cholangiocarcinoma	NCT04238715	Tasurgratinib	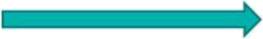
Malignant melanoma, Small cell lung cancer, Malignant thymoma	NCT03511222	Vorolanib	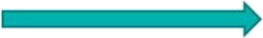
Renal cell carcinoma	NCT03511222	Vorolanib	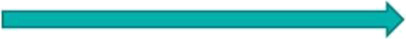
Cervical intraepithelial neoplasia	NCT04276688	Lopinavir/Ritonavir	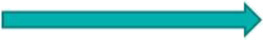
Neuroendocrine tumor	NCT01298999	Netazepide	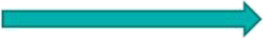

## Conclusion

In this review, we deeply discussed the key role played by urea moiety in the development of anticancer agents, outlying its presence in several approved antitumor drugs as well as in novel hits and drug candidates. The specific physico-chemical properties of urea moiety, and the possibility to modulate different targets by properly selecting the nitrogen substituents opens many possibilities in drug design. The urea functionality may be fundamental not only for drug-target interactions but also for improving the pharmacokinetic profile of a drug candidate. The present review is expected to provide useful insights in the design of urea-based compounds and would inspire the medicinal chemists for the development of clinically viable candidates. as well as new formulations for old urea-based drugs. Developing innovative pharmaceutical platforms is indeed a winning strategy to improve the pharmacological profile of already marketed drugs, also to bypass developability issues related to the urea moiety, when it is essential for the biological activity. Despite numerous advances, scientists are still trying to develop novel drugs that are useful in clinical oncology and able to overcome resistance to chemotherapy, which is mainly due to protein mutations, i.e., kinases or stress oxidation machinery dysregulation. As extensively herein discussed, urea derivatives can be a powerful tool for the discovery of new, effective therapeutics and hopefully inspire future research toward the development of viable strategies to fight cancer.

## References

[B1] Alex A. Millan D. S. Perez M. Wakenhut F. Whitlock G. A. (2011). Intramolecular hydrogen bonding to improve membrane permeability and absorption in beyond rule of five chemical space. Medchemcomm 2, 669. 10.1039/c1md00093d

[B2] Ali M. Chen H.-Y. Chiang Y.-F. Badary O. A. Hsia S.-M. Al-Hendy A. (2022). An evaluation of relugolix/estradiol/norethindrone acetate for the treatment of heavy menstrual bleeding associated with uterine fibroids in premenopausal women. Expert Opin. Pharmacother. 23, 421–429. 10.1080/14656566.2022.2030705 35068291 PMC8866208

[B3] André F. Ciruelos E. M. Juric D. Loibl S. Campone M. Mayer I. A. (2021). Alpelisib plus fulvestrant for PIK3CA-mutated, hormone receptor-positive, human epidermal growth factor receptor-2–negative advanced breast cancer: Final overall survival results from SOLAR-1. Ann. Oncol. 32, 208–217. 10.1016/j.annonc.2020.11.011 33246021

[B4] Asih P. R. Prikas E. Stefanoska K. Tan A. R. P. Ahel H. I. Ittner A. (2020). Functions of p38 MAP kinases in the central nervous system. Front. Mol. Neurosci. 13, 570586. 10.3389/fnmol.2020.570586 33013322 PMC7509416

[B5] Avendaño C. Menéndez J. C. (2015). “DNA alkylating agents,” in Medicinal chemistry of anticancer drugs (Netherlands: Elsevier), 197–241. 10.1016/B978-0-444-62649-3.00005-3

[B6] Ayala-Aguilera C. C. Valero T. Lorente-Macías Á. Baillache D. J. Croke S. Unciti-Broceta A. (2022). Small molecule kinase inhibitor drugs (1995–2021): Medical indication, pharmacology, and synthesis. J. Med. Chem. 65, 1047–1131. 10.1021/acs.jmedchem.1c00963 34624192

[B7] Ayati A. Moghimi S. Salarinejad S. Safavi M. Pouramiri B. Foroumadi A. (2020). A review on progression of epidermal growth factor receptor (EGFR) inhibitors as an efficient approach in cancer targeted therapy. Bioorg. Chem. 99, 103811. 10.1016/j.bioorg.2020.103811 32278207

[B8] Bajusz S. Csernus V. J. Janaky T. Bokser L. Fekete M. Schally A. V. (2009). New antagonists of LHRH II. Inhibition and potentiation of LHRH by closely related analogues. Int. J. Pept. Protein Res. 32, 425–435. 10.1111/j.1399-3011.1988.tb01373.x 2469662

[B9] Banerjee S. R. Pullambhatla M. Byun Y. Nimmagadda S. Green G. Fox J. J. (2010). 68 Ga-labeled inhibitors of prostate-specific membrane antigen (PSMA) for imaging prostate cancer. J. Med. Chem. 53, 5333–5341. 10.1021/jm100623e 20568777 PMC3341619

[B10] Barinka C. Byun Y. Dusich C. L. Banerjee S. R. Chen Y. Castanares M. (2008). Interactions between human glutamate carboxypeptidase II and urea-based inhibitors: Structural characterization. J. Med. Chem. 51, 7737–7743. 10.1021/jm800765e 19053759 PMC5516903

[B11] Barvian M. Boschelli D. H. Cossrow J. Dobrusin E. Fattaey A. Fritsch A. (2000). Pyrido [ 2 , 3- d ] pyrimidin-7-one inhibitors of cyclin-dependent kinases. J. Med. Chem. 43, 4606–4616. 10.1021/jm000271k 11101352

[B12] Bavetsias V. Linardopoulos S. (2015). Aurora kinase inhibitors: Current status and outlook. Front. Oncol. 5, 278. 10.3389/fonc.2015.00278 26734566 PMC4685048

[B13] Benešová M. Schäfer M. Bauder-Wüst U. Afshar-Oromieh A. Kratochwil C. Mier W. (2015). Preclinical evaluation of a tailor-made DOTA-conjugated PSMA inhibitor with optimized linker moiety for imaging and endoradiotherapy of prostate cancer. J. Nucl. Med. 56, 914–920. 10.2967/jnumed.114.147413 25883127

[B14] Bhuyan B. K. (1970). The action of streptozotocin on mammalian cells. Cancer Res. 30, 2017–2023. 5456086

[B15] Bi F. Qiu M. Chai X. Niu J. Ding Y. Bai Y. (2017). A multicenter phase II study of donafenib in patients with advanced hepatocellular carcinoma. J. Clin. Oncol. 35, e15682. 10.1200/JCO.2017.35.15_suppl.e15682

[B16] Blencowe P. Mark C. Andrew C. Tennyson E. Robert H. Ellen M. (2020a). Heterocyclic substituted ureas, for use against cancer. WO2020030925A1.

[B17] Blencowe P. Mark C. Tennyson E. Ellen M. Hollie M. Laurent R. (2020b).Thiazoleureas as anticancer agents. WO2020030924A1.

[B18] Boni J. Rubio-Perez C. López-Bigas N. Fillat C. de la Luna S. (2020). The DYRK family of kinases in cancer: Molecular functions and therapeutic opportunities. Cancers (Basel) 12, 2106–2126. 10.3390/cancers12082106 PMC7465136 32751160

[B19] Brentjens R. Saltz L. (2001). Islet cell tumors of the pancreas: The medical oncologist’s perspective. Surg. Clin. North Am. 81, 527–542. 10.1016/S0039-6109(05)70141-9 11459269

[B20] Bruce I. Akhlaq M. Bloomfield G. C. Budd E. Cox B. Cuenoud B. (2012). Development of isoform selective PI3-kinase inhibitors as pharmacological tools for elucidating the PI3K pathway. Bioorg. Med. Chem. Lett. 22, 5445–5450. 10.1016/j.bmcl.2012.07.042 22863202

[B21] Brünner N. A. Jan S. (2021). Urea derivatives for use in the treatment of subjects with elevated expression and/or activity of SRPK1. US2021251963A1.

[B22] Buchstaller H.-P. Dieter D. (2013). Piperidine urea derivatives.

[B23] Cadène M. Duranton J. North A. Tahar S.-M. Chignard M. Bieth J. G. (1997). Inhibition of neutrophil serine proteinases by suramin. J. Biol. Chem. 272, 9950–9955. 10.1074/jbc.272.15.9950 9092534

[B24] Cai D. Bo H. Chungang F. Yunhong J. (2019). 4-Phenylthiazole-2-amine derivative containing urea structure, and preparation method and application thereof. CN109456280A.

[B25] Catalano A. Iacopetta D. Sinicropi M. S. Franchini C. (2021). Diarylureas as antitumor agents. Appl. Sci. (Basel). 11, 374. 10.3390/app11010374 PMC7833385 33477901

[B26] Chao Q. Sprankle K. G. Grotzfeld R. M. Lai A. G. Carter T. A. Velasco A. M. (2009). Identification of N-(5-tert-butyl-isoxazol-3-yl)-N′-{4-[7-(2- morpholin-4-yl-ethoxy)imidazo-[2, 1-b] [1, 3]benzothiazol-2-yl]phenyl}urea dihydrochloride (AC220), a uniquely potent, selective, and efficacious FMS-like tyrosine kinase-3 (FLT3) inhibitor. J. Med. Chem. 52, 7808–7816. 10.1021/jm9007533 19754199

[B27] Chen Y. Pullambhatla M. Foss C. A. Byun Y. Nimmagadda S. Senthamizhchelvan S. (2011). 2-(3-{1-Carboxy-5-[(6-[18F]Fluoro-Pyridine-3-Carbonyl)-Amino]-Pentyl}-Ureido)-Pentanedioic acid, [18F]DCFPyL, a PSMA-based PET imaging agent for prostate cancer. Clin. Cancer Res. 17, 7645–7653. 10.1158/1078-0432.CCR-11-1357 22042970 PMC3243762

[B28] Cheng C. Ying-Jun Z. Bing L. Bo-Hua L. Yu C. Zhi-Xin C. (2018). Substituted urea derivatives and pharmaceutical uses thereof. US10065934B2.

[B29] Cheng C. Ying-Jun Z. Bing L. Bo-Hua L. Yu C. Zhi-Xin C. (2017). Substituted urea derivatives and pharmaceutical uses thereof which provides substituted urea derivatives, and its stereoisomer, geometric isomer, tautomer, nitroxide, hydrate, solvate, metabolism product, ester, and pharmaceutically accepted salt or its p. TW201706256A.

[B30] Chuu C.-P. Kokontis J. M. Hiipakka R. A. Fukuchi J. Lin H.-P. Lin C.-Y. (2011). Androgens as therapy for androgen receptor-positive castration-resistant prostate cancer. J. Biomed. Sci. 18, 63. 10.1186/1423-0127-18-63 21859492 PMC3170584

[B31] Cohen P. Cross D. Jänne P. A. (2021). Kinase drug discovery 20 years after imatinib: Progress and future directions. Nat. Rev. Drug Discov. 20, 551–569. 10.1038/s41573-021-00195-4 34002056 PMC8127496

[B32] Collina S. Daniela R. Pasquale L. Giacomo R. Roberta L. Guido C. (2022). Substituted vinyl piperazine-piperidine urea derivatives as anticancer agents. EP21201359.

[B33] Colvin M. Brundrett R. B. Cowens W. Jardine I. Ludlum D. B. (1976). A chemical basis for the antitumor activity of chloroethylnitrosoureas. Biochem. Pharmacol. 25, 695–699. 10.1016/0006-2952(76)90246-X 945062

[B34] Cui Y. (2013). Hydrotropic solubilization by urea derivatives: A molecular dynamics simulation study. J. Pharm. (Cairo). 2013, 1–15. –15. 10.1155/2013/791370 PMC4590820 26555993

[B35] Della Puppa A. Rossetto M. Ciccarino P. Denaro L. Rotilio A. D’Avella D. (2011). Carmustine wafer implantation when surgical cavity is communicating with cerebral ventricles: Technical considerations on a clinical series. World Neurosurg. x. 76, 156–159. 10.1016/j.wneu.2010.10.024 21839967

[B36] Dhillon S. (2018). Regorafenib: A review in metastatic colorectal cancer. Drugs 78, 1133–1144. 10.1007/s40265-018-0938-y 29943375

[B37] Dogan S. D. Betul A. M. Kubra K. A. A. Ruveyde K. (2021). Synthesis of urea derivatives which have p38 mapk inhibition and anticancer efficacy. WO2021133319A1.

[B38] Dutta A. S. Furr B. J. A. Giles M. B. Valcaccia B. (1978). Synthesis and biological activity of highly active .alpha.-aza analogs of luliberin. J. Med. Chem. 21, 1018–1024. 10.1021/jm00208a004 364060

[B39] Eichhorst S. T. Krueger A. Müerköster S. Fas S. C. Golks A. Gruetzner U. (2004). Suramin inhibits death receptor–induced apoptosis *in vitro* and fulminant apoptotic liver damage in mice. Nat. Med. 10, 602–609. 10.1038/nm1049 15146177

[B40] Evans J. C. Malhotra M. Cryan J. F. O’Driscoll C. M. (2016). The therapeutic and diagnostic potential of the prostate specific membrane antigen/glutamate carboxypeptidase II (PSMA/GCPII) in cancer and neurological disease. Br. J. Pharmacol. 173, 3041–3079. 10.1111/bph.13576 27526115 PMC5056232

[B41] Federman N. McDermott R. (2019). Larotrectinib, a highly selective tropomyosin receptor kinase (TRK) inhibitor for the treatment of TRK fusion cancer. Expert Rev. Clin. Pharmacol. 12, 931–939. 10.1080/17512433.2019.1661775 31469968

[B42] Feng Y. Li M. Wang B. Zheng Y. G. (2010). Discovery and mechanistic study of a class of protein arginine methylation inhibitors. J. Med. Chem. 53, 6028–6039. 10.1021/jm100416n 20666457

[B43] Firsching A. Nickel P. Allolio B. Mora P. (1995). Antiproliferative and angiostatic activity of suramin analogues. Cancer Res. 55, 4957–4961. 7585536

[B44] Flynn D. L. Kaufman M. D. Petillo P. A. (2012). Dihydronaphthyridines and related compounds useful as kinase inhibitors for the treatment of proliferative diseases. US8461179B1.

[B45] Fukushima N. Minami Y. Kakiuchi S. Kuwatsuka Y. Hayakawa F. Jamieson C. (2016). Small‐molecule Hedgehog inhibitor attenuates the leukemia‐initiation potential of acute myeloid leukemia cells. Cancer Sci. 107, 1422–1429. 10.1111/cas.13019 27461445 PMC5084664

[B46] Furet P. Caravatti G. Guagnano V. Lang M. Meyer T. Schoepfer J. (2008). Entry into a new class of protein kinase inhibitors by pseudo ring design. Bioorg. Med. Chem. Lett. 18, 897–900. 10.1016/j.bmcl.2007.12.041 18248988

[B47] Furet P. Guagnano V. Fairhurst R. A. Imbach-Weese P. Bruce I. Knapp M. (2013). Discovery of NVP-BYL719 a potent and selective phosphatidylinositol-3 kinase alpha inhibitor selected for clinical evaluation. Bioorg. Med. Chem. Lett. 23, 3741–3748. 10.1016/j.bmcl.2013.05.007 23726034

[B48] Ganis P. Avitabile G. Benedetti E. Pedone C. Goodman M. (1970). Crystal and molecular structure of N, N ′-Diethyl- N, N ′-Diphenylurea. Proc. Natl. Acad. Sci. U. S. A. 67, 426–433. 10.1073/pnas.67.1.426 16591865 PMC283222

[B49] Garcia-Horton A. Yee K. W. (2020). Quizartinib for the treatment of acute myeloid leukemia. Expert Opin. Pharmacother. 21, 2077–2090. 10.1080/14656566.2020.1801637 32772726

[B50] Gaulton A. Bellis L. J. Bento A. P. Chambers J. Davies M. Hersey A. (2012). ChEMBL: A large-scale bioactivity database for drug discovery. Nucleic Acids Res. 40, D1100–D1107. 10.1093/nar/gkr777 21948594 PMC3245175

[B51] Ghosh A. K. Brindisi M. (2020). Urea derivatives in modern drug discovery and medicinal chemistry. J. Med. Chem. 63, 2751–2788. 10.1021/acs.jmedchem.9b01541 31789518 PMC7266097

[B52] Gnewuch C. T. Sosnovsky G. (1997). A critical appraisal of the evolution of N -nitrosoureas as anticancer drugs. Chem. Rev. 97, 829–1014. 10.1021/cr941192h 11848890

[B53] Goldsmith S. R. Lovell A. R. Schroeder M. A. (2019). Glasdegib for the treatment of adult patients with newly diagnosed acute myeloid leukemia or high-grade myelodysplastic syndrome who are elderly or otherwise unfit for standard induction chemotherapy. Drugs Today (Barc). 55, 545. 10.1358/dot.2019.55.9.3020160 31584572

[B153] Gömeç M. Yulak F. Gezegen H. Özkaraca M. Sayin K. Ataseven H. (2022). Synthesis of diaryl urea derivatives and evaluation of their antiproliferative activities in colon adenocarcinoma. J. Mol. Struct. 1254. 10.1016/j.molstruc.2021.132318

[B54] Guagnano V. Furet P. Spanka C. Bordas V. Le Douget M. Stamm C. (2011). Discovery of 3-(2, 6-Dichloro-3, 5-dimethoxy-phenyl)-1-{6-[4-(4-ethyl- piperazin-1-yl)-phenylamino]-pyrimidin-4-yl}-1-methyl-urea (NVP-BGJ398), A potent and selective inhibitor of the fibroblast growth factor receptor family of receptor tyrosine kinase. J. Med. Chem. 54, 7066–7083. 10.1021/jm2006222 21936542

[B55] Hamidi S. Boucher A. Lemieux B. Rondeau G. Lebœuf R. Ste-Marie L.-G. (2022). Lenvatinib therapy for advanced thyroid cancer: Real-life data on safety, efficacy, and some rare side effects. J. Endocr. Soc. 6, bvac048. 10.1210/jendso/bvac048 35475024 PMC9032633

[B56] Hayes M. T. Bartley J. Parsons P. G. Eaglesham G. K. Prakash A. S. (1997). Mechanism of action of fotemustine, a new chloroethylnitrosourea anticancer agent: Evidence for the formation of two DNA-reactive intermediates contributing to cytotoxicity. Biochemistry 36, 10646–10654. 10.1021/bi970791q 9271495

[B57] Hensey C. E. Boscoboinik D. Azzi A. (1989). Suramin, an anti-cancer drug, inhibits protein kinase C and induces differentiation in neuroblastoma cell clone NB2A. FEBS Lett. 258, 156–158. 10.1016/0014-5793(89)81639-4 2591531

[B58] Henß L. Beck S. Weidner T. Biedenkopf N. Sliva K. Weber C. (2016). Suramin is a potent inhibitor of Chikungunya and Ebola virus cell entry. Virol. J. 13, 149. 10.1186/s12985-016-0607-2 27581733 PMC5007819

[B59] Heo Y.-A. Syed Y. Y. (2018). Regorafenib: A review in hepatocellular carcinoma. Drugs 78, 951–958. 10.1007/s40265-018-0932-4 29915898

[B60] Herbig M. E. Evers D.-H. (2013). Correlation of hydrotropic solubilization by urea with logD of drug molecules and utilization of this effect for topical formulations. Eur. J. Pharm. Biopharm. 85, 158–160. 10.1016/j.ejpb.2013.06.022 23958327

[B61] Holderfield M. Nagel T. E. Stuart D. D. (2014). Mechanism and consequences of RAF kinase activation by small-molecule inhibitors. Br. J. Cancer 111, 640–645. 10.1038/bjc.2014.139 24642617 PMC4134487

[B62] Hosokawa M. Dolci W. Thorens B. (2001). Differential sensitivity of GLUT1- and GLUT2-expressing β cells to streptozotocin. Biochem. Biophys. Res. Commun. 289, 1114–1117. 10.1006/bbrc.2001.6145 11741307

[B154] Hou S. Liang S. Zhang C. Han Y. Liang J. Hu H. (2021). Design, synthesis and anticancer activity of a new series of n-aryl-n′-[4-(Pyridin-2-ylmethoxy)benzyl]urea derivatives. Molecules 26, 3496. 10.3390/molecules26123496 34201326 PMC8226862

[B63] Huirne J. A. Lambalk C. B. (2001). Gonadotropin-releasing-hormone-receptor antagonists. Lancet 358, 1793–1803. 10.1016/S0140-6736(01)06797-6 11734258

[B64] Ishikawa M. Hashimoto Y. (2011). Improvement in aqueous solubility in small molecule drug discovery programs by disruption of molecular planarity and symmetry. J. Med. Chem. 54, 1539–1554. 10.1021/jm101356p 21344906

[B65] Jiang G. Stalewski J. Galyean R. Dykert J. Schteingart C. Broqua P. (2001). GnRH antagonists: A new generation of long acting analogues incorporating p-Ureido-phenylalanines at positions 5 and 6. J. Med. Chem. 44, 453–467. 10.1021/jm0003900 11462984

[B66] Jianian C. Xianfu W. Guangji Z. Xiaobo F. Xingcan S. Dewen W. (2014). Quinazolinyl-aryl urea derivatives with antitumor function and application thereof. CN104292170A.

[B155] Jin P.-R. Ta Y.-N. N. Chen I.-T. Yu Y.-N. Hsieh H. T. Nguyen V.-A. T. (2021). Cinchona Alkaloid-Inspired Urea-Containing Autophagy Inhibitor Shows Single-Agent Anticancer Efficacy. J. Med. Chem. 64, 14513–14525. 10.1021/acs.jmedchem.1c01036 34558909

[B67] Kakuguchi W. Nomura T. Kitamura T. Otsuguro S. Matsushita K. Sakaitani M. (2018). Suramin, screened from an approved drug library, inhibits HuR functions and attenuates malignant phenotype of oral cancer cells. Cancer Med. 7, 6269–6280. 10.1002/cam4.1877 30449075 PMC6308099

[B68] Kannaiyan R. Mahadevan D. (2018). A comprehensive review of protein kinase inhibitors for cancer therapy. Expert Rev. Anticancer Ther. 18, 1249–1270. 10.1080/14737140.2018.1527688 30259761 PMC6322661

[B69] Karoulia Z. Gavathiotis E. Poulikakos P. I. (2017). New perspectives for targeting RAF kinase in human cancer. Nat. Rev. Cancer 17, 676–691. 10.1038/nrc.2017.79 28984291 PMC6000833

[B151] Khalifa S. A. M. Elias N. Farag M. A. Chen L. Saeed A. Hegazy M.-E. F. (2019). Marine Natural Products: A Source of Novel Anticancer Drugs. Mar. Drugs 17, 491. 10.3390/md17090491 PMC6780632 31443597

[B104] Kim J. Nguyen M. N. Enlow E. Ong W. Z. Nowak P. W. Feutrill J. T. (2013). Urea derivatives and uses thereof. WO/2014/201127.

[B70] Kim I. S. Sik K. H. (2018). N-N-aroylureas derivatives preparation method thereof and pharmaceutical compositions for the prevention and treatment of cancer containing the same as an active ingredient. KR101849775B1.

[B71] Kim Seong H. (2013). A Cancer sensitizer comprising phenylurea derivatives or salts thereof. KR101332830B1.

[B72] Koç A. Wheeler L. J. Mathews C. K. Merrill G. F. (2004). Hydroxyurea arrests DNA replication by a mechanism that preserves basal dNTP pools. J. Biol. Chem. 279, 223–230. 10.1074/jbc.M303952200 14573610

[B73] Kohn K. (1977). Interstrand cross-linking of DNA by 1, 3-bis(2-chloroethyl)-1-nitrosourea and other 1-(2-haloethyl)-1-nitrosoureas. Cancer Res. 37, 1450–1454. 851960

[B74] Korbecki J. Kupnicka P. Chlubek M. Gorący J. Gutowska I. Baranowska-Bosiacka I. (2022). CXCR2 receptor: Regulation of expression, signal transduction, and involvement in cancer. Int. J. Mol. Sci. 23, 2168. 10.3390/ijms23042168 35216283 PMC8878198

[B75] Koul H. K. Pal M. Koul S. (2013). Role of p38 MAP kinase signal transduction in solid tumors. Genes Cancer 4, 342–359. 10.1177/1947601913507951 24349632 PMC3863344

[B76] Kozikowski A. P. Nan F. Conti P. Zhang J. Ramadan E. Bzdega T. (2001). Design of remarkably simple, yet potent urea-based inhibitors of glutamate carboxypeptidase II (NAALADase). J. Med. Chem. 44, 298–301. 10.1021/jm000406m 11462970

[B77] Kubo K. Sakai T. Nagao R. Fujiwara Y. Isoe T. Hasegawa K. (2002). Quinoline derivative having azolyl group and quinazoline derivative. US20030087907A1.

[B78] Kumar N. Lal N. Nemaysh V. Luthra P. M. (2020). Design, synthesis, DNA binding studies and evaluation of anticancer potential of novel substituted biscarbazole derivatives against human glioma U87 MG cell line. Bioorg. Chem. 100, 103911. 10.1016/j.bioorg.2020.103911 32502918

[B79] Kumari S. Carmona A. V. Tiwari A. K. Trippier P. C. (2020). Amide bond bioisosteres: Strategies, synthesis, and successes. J. Med. Chem. 63, 12290–12358. 10.1021/acs.jmedchem.0c00530 32686940 PMC7666045

[B80] Kuo S.-C. Wang Y.-M. Ho Y.-J. Chang T.-Y. Lai Z.-Z. Tsui P.-Y. (2016). Suramin treatment reduces chikungunya pathogenesis in mice. Antivir. Res. 134, 89–96. 10.1016/j.antiviral.2016.07.025 27577529

[B81] Lee C. Hyun-Mo Y. Hojin C. Dohoon K. Soyoung K. Nina H. (2013). Novel compounds for selective histone deacetylase inhibitors, and pharmaceutical composition comprising the same. US2016083354A1.

[B152] Leggans E. K. Duncan K. K. Barker T. J. Schleicher K. D. Boger D. L. (2013). A remarkable series of vinblastine analogues displaying enhanced activity and an unprecedented tubulin binding steric tolerance: C20′ urea derivatives. J. Med. Chem. 56, 628–639. 10.1021/jm3015684 23244701 PMC3574233

[B82] Li X. Qiu M. Wang S. Zhu H. Feng B. Zheng L. (2020). A Phase I dose-escalation, pharmacokinetics and food-effect study of oral donafenib in patients with advanced solid tumours. Cancer Chemother. Pharmacol. 85, 593–604. 10.1007/s00280-020-04031-1 32008115

[B83] Liang X. Yang Q. Wu P. He C. Yin L. Xu F. (2021). The synthesis review of the approved tyrosine kinase inhibitors for anticancer therapy in 2015–2020. Bioorg. Chem. 113, 105011. 10.1016/j.bioorg.2021.105011 34091289

[B84] Lim S. Ryu J. H. Lm C. Yim C. B. (2003). Synthesis and cytotoxicity of new 3-alkyl-1-(1-methyl-2-phenylethyl)ureas related to ceramide. Arch. Pharm. Res. 26, 270–274. 10.1007/BF02976954 12735683

[B85] Linciano P. Nasti R. Listro R. Amadio M. Pascale A. Potenza D. (2022). Chiral 2‐phenyl‐3‐hydroxypropyl esters as PKC‐alpha modulators: HPLC enantioseparation, NMR absolute configuration assignment, and molecular docking studies. Chirality 34, 498–513. 10.1002/chir.23406 34962318

[B86] Lortie F. Boileau S. Bouteiller L. (2003). N, N-disubstituted ureas: Influence of substituents on the formation of supramolecular polymers. Chem. Eur. J. 9, 3008–3014. 10.1002/chem.200304801 12833282

[B87] Lowinger T. Riedl B. Dumas J. Smith R. (2002). Design and discovery of small molecules targeting raf-1 kinase. Curr. Pharm. Des. 8, 2269–2278. 10.2174/1381612023393125 12369855

[B88] Malacrida A. Cavalloro V. Martino E. Costa G. Ambrosio F. A. Alcaro S. (2021). Anti-multiple myeloma potential of secondary metabolites from Hibiscus sabdariffa—Part 2. Molecules 26, 6596. 10.3390/molecules26216596 34771006 PMC8588054

[B89] Martino E. Casamassima G. Castiglione S. Cellupica E. Pantalone S. Papagni F. (2018). Vinca alkaloids and analogues as anti-cancer agents: Looking back, peering ahead. Bioorg. Med. Chem. Lett. 28, 2816–2826. 10.1016/j.bmcl.2018.06.044 30122223

[B90] Martino E. Della Volpe S. Terribile E. Benetti E. Sakaj M. Centamore A. (2017). The long story of camptothecin: From traditional medicine to drugs. Bioorg. Med. Chem. Lett. 27, 701–707. 10.1016/j.bmcl.2016.12.085 28073672

[B91] McCain D. F. Wu L. Nickel P. Kassack M. U. Kreimeyer A. Gagliardi A. (2004). Suramin derivatives as inhibitors and activators of protein-tyrosine phosphatases. J. Biol. Chem. 279, 14713–14725. 10.1074/jbc.M312488200 14734566

[B92] Menaa F. (2013). Latest approved therapies for metastatic melanoma: What comes next? J. Skin. Cancer 2013, 1–10. 10.1155/2013/735282 PMC3595667 23533766

[B93] Moarbess G. Deleuze-Masquefa C. Bonnard V. Gayraud-Paniagua S. Vidal J. R. Bressolle F. (2008). *In vitro* and *in vivo* anti-tumoral activities of imidazo[1, 2-a]quinoxaline, imidazo[1, 5-a]quinoxaline, and pyrazolo[1, 5-a]quinoxaline derivatives. Bioorg. Med. Chem. 16 (13), 6601–6610. 10.1016/j.bmc.2008.05.022 18513976

[B94] Modi S. J. Anshuly T. Madhvarao K. V. (2021). A rational Drug Design based identification of orally bioavailable 1,5-disubstituted naphthalene compounds as potent VEGFR-2 inhibitors. AU2021103375A4.

[B95] Modi S. J. Kulkarni V. M. (2019). Vascular endothelial growth factor receptor (VEGFR-2)/KDR inhibitors: Medicinal chemistry perspective. Med. Drug Discov. 2, 100009. 10.1016/j.medidd.2019.100009

[B96] Mohammadi M. (1998). Crystal structure of an angiogenesis inhibitor bound to the FGF receptor tyrosine kinase domain. EMBO J. 17, 5896–5904. 10.1093/emboj/17.20.5896 9774334 PMC1170917

[B97] Motzer R. Alekseev B. Rha S.-Y. Porta C. Eto M. Powles T. (2021). Lenvatinib plus pembrolizumab or everolimus for advanced renal cell carcinoma. N. Engl. J. Med. 384, 1289–1300. 10.1056/NEJMoa2035716 33616314

[B98] Motzer R. J. Hutson T. E. Glen H. Michaelson M. D. Molina A. Eisen T. (2015). Lenvatinib, everolimus, and the combination in patients with metastatic renal cell carcinoma: A randomised, phase 2, open-label, multicentre trial. Lancet Oncol. 16, 1473–1482. 10.1016/S1470-2045(15)00290-9 26482279

[B99] Nakajima M. DeChavigny A. Johnson C. E. Hamada J. I. Stein C. A. Nicolson G. L. (1991). Suramin: A potent inhibitor of melanoma heparanase and invasion. J. Biol. Chem. 266, 9661–9666. 10.1016/s0021-9258(18)92871-1 2033058

[B100] Nakamura K. Taguchi E. Miura T. Yamamoto A. Takahashi K. Bichat F. (2006). KRN951, a highly potent inhibitor of vascular endothelial growth factor receptor tyrosine kinases, has antitumor activities and affects functional vascular properties. Cancer Res. 66, 9134–9142. 10.1158/0008-5472.CAN-05-4290 16982756

[B101] Nasser N. J. (2022). Androgen flare after LHRH initiation is the side effect that makes most of the beneficial effect when it coincides with radiation therapy for prostate cancer. Cancers (Basel). 14, 1959. 10.3390/cancers14081959 35454866 PMC9029515

[B102] Nestor J. J. Tahilramani R. Ho T. L. Goodpasture J. C. Vickery B. H. Ferrandon P. (1992). Potent gonadotropin releasing hormone antagonists with low histamine-releasing activity. J. Med. Chem. 35, 3942–3948. 10.1021/jm00099a023 1279174

[B103] Okamoto K. Ikemori-kawada M. Jestel A. Funahashi Y. Matsushima T. Tsuruoka A. (2015). Distinct binding mode of multikinase inhibitor lenvatinib revealed by biochemical characterization. ACS Med. Chem. Lett. 6, 89–94. 10.1021/ml500394m 25589937 PMC4291723

[B105] Osswald H. Youssef M. (1979). Suramin enhancement of the chemotherapeutic actions of cyclophosphamide or adriamycin of intramuscularly-implanted Ehrlich carcinoma. Cancer Lett. 6, 337–343. 10.1016/S0304-3835(79)80091-9 455271

[B156] Özgeriş F. B. Kaci F. N. Özgeriş B. Görmez A. (2022). The synthesis of unsymmetrical urea from substituted phenethylamine and the investigation of its antibacterial, anticancer, and antioxidant properties. Biointerface Res. Appl. Chem. 12, 7052–7063. 10.33263/BRIAC125.70527063

[B106] Pak E. Segal R. A. (2016). Hedgehog signal transduction: Key players, oncogenic drivers, and cancer therapy. Dev. Cell 38, 333–344. 10.1016/j.devcel.2016.07.026 27554855 PMC5017307

[B107] Parveen N. Lin Y.-L. Khan M. I. Chou R.-H. Sun C.-M. Yu C. (2020). Suramin derivatives play an important role in blocking the interaction between FGF1 and FGFRD2 to inhibit cell proliferation. Eur. J. Med. Chem. 206, 112656. 10.1016/j.ejmech.2020.112656 32827875

[B108] Parveen N. Lin Y. L. Chou R. H. Sun C. M. Yu C. (2022). Synthesis of novel suramin analogs with anti-proliferative activity via FGF1 and FGFRD2 blockade. Front. Chem. 9, 764200–764220. 10.3389/fchem.2021.764200 35047478 PMC8763243

[B109] Pastorino S. Riondato M. Uccelli L. Giovacchini G. Giovannini E. Duce V. (2020). Toward the discovery and development of PSMA targeted inhibitors for nuclear medicine applications. Curr. Radiopharm. 13, 63–79. 10.2174/1874471012666190729151540 31362683 PMC7509769

[B110] Patel H. K. Grotzfeld R. M. Lai A. G. Mehta S. A. Milanov Z. V. Chao Q. (2009). Arylcarboxyamino-substituted diaryl ureas as potent and selective FLT3 inhibitors. Bioorg. Med. Chem. Lett. 19, 5182–5185. 10.1016/j.bmcl.2009.07.024 19646870

[B111] Persson B.-E. Kold Olesen T. Jensen J.-K. (2009). Degarelix: A new approach for the treatment of prostate cancer. Neuroendocrinology 90, 235–244. 10.1159/000228832 19602868

[B112] Riedl B. Lowinger T.B Bankston D. Barbosa J. Brittelli D.R. Carlson R. (2001). Potent Raf kinase inhibitors from the diphenylurea class Structure activity relationships. Proc. Am. Assoc. Cancer Res. Annu. Meet. 42, 923.

[B113] Rowe S. P. Gage K. L. Faraj S. F. Macura K. J. Cornish T. C. Gonzalez-Roibon N. (2015). 18 F-DCFBC PET/CT for PSMA-based detection and characterization of primary prostate cancer. J. Nucl. Med. 56, 1003–1010. 10.2967/jnumed.115.154336 26069305 PMC4659400

[B114] Rui M. Nasti R. Bignardi E. Della Volpe S. Rossino G. Rossi D. (2017). PKC in regenerative therapy: New insights for old targets. Pharmaceuticals 10, 46. 10.3390/ph10020046 PMC5490403 28524095

[B115] Sabbah D. A. Hajjo R. Sweidan K. (2020). Review on epidermal growth factor receptor (EGFR) structure, signaling pathways, interactions, and recent updates of EGFR inhibitors. Curr. Top. Med. Chem. 20, 815–834. 10.2174/1568026620666200303123102 32124699

[B116] Saitoh F. Hiroshi N. Yuji K. Tsutomu S. (2018). Novel tetrahydronaphthyl urea derivatives. WO2018199166A1.

[B117] Salem A. K. Elsaid A. S. A. M. (2020). JNK inhibitors as anticancer agents. WO2020263989A1.

[B118] Salgia N. J. Zengin Z. B. Pal S. K. (2020). Tivozanib in renal cell carcinoma: A new approach to previously treated disease. Ther. Adv. Med. Oncol. 12, 175883592092381. 10.1177/1758835920923818 PMC7249546 32547647

[B119] Sawa A. Hideki N. Kawahata R. Sawa W. Iwata M. Nagao Y. (2022). Cyclic urea derivatives. WO2022059778A1.

[B120] Shibuya M. (2011). Vascular endothelial growth factor (vegf) and its receptor (VEGFR) signaling in angiogenesis: A crucial target for anti- and pro-angiogenic therapies. Genes Cancer 2, 1097–1105. 10.1177/1947601911423031 22866201 PMC3411125

[B121] Shore N. D. Saad F. Cookson M. S. George D. J. Saltzstein D. R. Tutrone R. (2020). Oral relugolix for androgen-deprivation therapy in advanced prostate cancer. N. Engl. J. Med. 382, 2187–2196. 10.1056/NEJMoa2004325 32469183

[B122] Siddig L. A. Khasawneh M. A. Samadi A. Saadeh H. Abutaha N. Wadaan M. A. (2021). Synthesis of novel thiourea-/urea-benzimidazole derivatives as anticancer agents. Open Chem. 19, 1062–1073. 10.1515/chem-2021-0093

[B123] Smith B. D. Kaufman M. D. Lu W. P. Gupta A. Leary C. B. Wise S. C. (2019). Ripretinib (DCC-2618)Is a switch control kinase inhibitor of a broad spectrum of oncogenic and drug-resistant KIT and PDGFRA variants. Cancer Cell 35, 738–751.e9. e9. 10.1016/j.ccell.2019.04.006 31085175

[B124] Smith R. A. Barbosa J. Blum C. L. Bobko M. A. Caringal Y. V. Dally R. (2001). Discovery of heterocyclic ureas as a new class of raf kinase inhibitors: Identification of a second generation lead by a combinatorial chemistry approach. Bioorg. Med. Chem. Lett. 11, 2775–2778. 10.1016/S0960-894X(01)00571-6 11591521

[B125] Spivak J. L. Hasselbalch H. (2011). Hydroxycarbamide: A user’s guide for chronic myeloproliferative disorders. Expert Rev. Anticancer Ther. 11, 403–414. 10.1586/era.11.10 21417854

[B126] Springer C. J. Alfonso M. R. Romina G. Dan N.-D. Ion N.-D. (2013). 1-(5-tert-butyl-2-aryl-pyrazol-3-yl)-3-[2-fluoro-4-[(3-oxo-4h-pyrido[2,3-b]pyrazin-8-yl)oxy]phenyl]urea derivatives as Raf inhibitors for the treatment of cancer. US2016355510A1.

[B127] Steverding D. (2010). The development of drugs for treatment of sleeping sickness: A historical review. Parasit. Vectors 3, 15. 10.1186/1756-3305-3-15 20219092 PMC2848007

[B128] Tan C. W. Sam I.-C. Chong W. L. Lee V. S. Chan Y. F. (2017). Polysulfonate suramin inhibits Zika virus infection. Antivir. Res. 143, 186–194. 10.1016/j.antiviral.2017.04.017 28457855

[B129] Thompson L. Donald C. M. (2020). Glasdegib: A novel hedgehog pathway inhibitor for acute myeloid leukemia. J. Adv. Pract. Oncol. 11, 196–200. 10.6004/jadpro.2020.11.2.8 33532119 PMC7848813

[B130] Tong W. P. Ludlum D. B. (1978). Mechanism of action of the nitrosoureas—1. Biochem. Pharmacol. 27, 77–81. 10.1016/0006-2952(78)90259-9 619908

[B131] Trapp J. Meier R. Hongwiset D. Kassack M. U. Sippl W. Jung M. (2007). Structure–activity studies on suramin analogues as inhibitors of NAD+-Dependent histone deacetylases (sirtuins). ChemMedChem 2, 1419–1431. 10.1002/cmdc.200700003 17628866

[B132] Van Poppel H. (2010). Evaluation of degarelix in the management of prostate cancer. Cancer Manag. Res. 39–52. 10.2147/CMAR.S8841 PMC3004563 21188095

[B133] Varghese R. Dalvi Y. B. (2021). Natural products as anticancer agents. Curr. Drug Targets 22, 1272–1287. 10.2174/1389450121999201230204526 33390130

[B134] Vavra J. J. Deboer C. Dietz A. Hanka L. J. Sokolski W. T. (1960). Streptozotocin, a new antibacterial antibiotic. Antibiot. Annu. 7, 230–235. 13841501

[B135] Villalona-Calero M. A. Wientjes M. G. Otterson G. A. Kanter S. Young D. Murgo A. J. (2003). Phase I study of low-dose suramin as a chemosensitizer in patients with advanced non-small cell lung cancer. Clin. Cancer Res. 9, 3303–3311. Available at: http://www.ncbi.nlm.nih.gov/pubmed/12960116. 12960116

[B136] Wainwright M. (2010). Dyes, trypanosomiasis and DNA: A historical and critical review. Biotech. Histochem. 85, 341–354. 10.3109/10520290903297528 21080764

[B137] Wan P. T. C. Garnett M. J. Roe S. M. Lee S. Niculescu-Duvaz D. Good V. M. (2004). Mechanism of activation of the RAF-ERK signaling pathway by oncogenic mutations of B-RAF. Cell 116, 855–867. 10.1016/S0092-8674(04)00215-6 15035987

[B138] Wang S. Stephen P. Mingfeng Y. (2018). 5-(pyrimidin-4-yl)thiazol-2-yl urea derivatives as therapeutic agents. US11325900B2.

[B139] Wang X. Bove A. M. Simone G. Ma B. (2020). Molecular bases of VEGFR-2-mediated physiological function and pathological role. Front. Cell Dev. Biol. 8, 599281. 10.3389/fcell.2020.599281 33304904 PMC7701214

[B140] Wiedemar N. Hauser D. A. Mäser P. (2020). 100 years of suramin. Antimicrob. Agents Chemother. 64, e01168–19. 10.1128/AAC.01168-19 PMC7038244 31844000

[B141] Wilhelm S. Carter C. Lynch M. Lowinger T. Dumas J. Smith R. A. (2006). Discovery and development of sorafenib: A multikinase inhibitor for treating cancer. Nat. Rev. Drug Discov. 5, 835–844. 10.1038/nrd2130 17016424

[B142] Wilhelm S. M. Carter C. Tang L. Wilkie D. McNabola A. Rong H. (2004). BAY 43-9006 exhibits broad spectrum oral antitumor activity and targets the RAF/MEK/ERK pathway and receptor tyrosine kinases involved in tumor progression and angiogenesis. Cancer Res. 64, 7099–7109. 10.1158/0008-5472.CAN-04-1443 15466206

[B143] Wilhelm S. M. Dumas J. Adnane L. Lynch M. Carter C. A. Schütz G. (2011). Regorafenib (bay 73-4506): A new oral multikinase inhibitor of angiogenic, stromal and oncogenic receptor tyrosine kinases with potent preclinical antitumor activity. Int. J. Cancer 129, 245–255. 10.1002/ijc.25864 21170960

[B144] Yamamuro S. Takahashi M. Satomi K. Sasaki N. Kobayashi T. Uchida E. (2021). Lomustine and nimustine exert efficient antitumor effects against glioblastoma models with acquired temozolomide resistance. Cancer Sci. 112 (11), 4736–4747. 10.1111/cas.15141 34536314 PMC8586660

[B145] Yan M. Wang C. He B. Yang M. Tong M. Long Z. (2016). Aurora-A kinase: A potent oncogene and target for cancer therapy. Med. Res. Rev. 36, 1036–1079. 10.1002/med.21399 27406026

[B146] Yang J. C. H. Brose M. S. Castro G. Kim E. S. Lassen U. N. Leyvraz S. (2022). Rationale and design of ON-TRK: A novel prospective non-interventional study in patients with TRK fusion cancer treated with larotrectinib. BMC Cancer 22, 625. 10.1186/s12885-022-09687-x 35672677 PMC9171956

[B147] Yang J. Nie J. Ma X. Wei Y. Peng Y. Wei X. (2019). Targeting PI3K in cancer: Mechanisms and advances in clinical trials. Mol. Cancer 18, 26. 10.1186/s12943-019-0954-x 30782187 PMC6379961

[B148] Zhang D. (2012). Urea derivatives as kinase inhibitors. US2012077851A1.

[B149] Zhang S. Shuqiang S. Yuting T. (2012). N-substituted phenyl-N’-substituted heterocyclic urea compound and application thereof as anticancer medicament. WO2014040243A1.

[B150] Zhu H. Zefeng W. Pengfei W. Lu S. (2017). Design and synthesis of diphenyl urea derivative antitumor compounds containing pyrazol framework. CN107118157A.

